# Core and auxiliary functions of one-carbon metabolism in *Pseudomonas putida* exposed by a systems-level analysis of transcriptional and physiological responses

**DOI:** 10.1128/msystems.00004-23

**Published:** 2023-06-05

**Authors:** Justine Turlin, Òscar Puiggené, Stefano Donati, Nicolas T. Wirth, Pablo I. Nikel

**Affiliations:** 1 The Novo Nordisk Foundation Center for Biosustainability, Technical University of Denmark, Kongens Lyngby, Denmark; Medical Research Council Toxicology Unit, Cambridge, United Kingdom

**Keywords:** *Pseudomonas putida*, systems biology, methanol, formaldehyde, formate, carbon metabolism, transcriptional analysis

## Abstract

The soil bacterium *Pseudomonas putida* is a robust biomanufacturing host that assimilates a broad range of substrates while efficiently coping with adverse environmental conditions. *P. putida* is equipped with functions related to one-carbon (C1) compounds (e.g. methanol, formaldehyde, and formate) oxidation—yet pathways to assimilate these carbon sources are largely absent. In this work, we adopted a systems-level approach to study the genetic and molecular basis of C1 metabolism in *P. putida*. RNA sequencing identified two oxidoreductases, encoded by *PP_0256* and *PP_4596*, transcriptionally active in the presence of formate. Quantitative physiology of deletion mutants revealed growth defects at high formate concentrations, pointing to an important role of these oxidoreductases in C1 tolerance. Moreover, we describe a concerted detoxification process for methanol and formaldehyde, the C1 intermediates upstream formate. Alcohol oxidation to highly-reactive formaldehyde by PedEH and other broad-substrate-range dehydrogenases underpinned the (apparent) suboptimal methanol tolerance of *P. putida*. Formaldehyde was mostly processed by a glutathione-dependent mechanism encoded in the *frmAC* operon, and thiol-independent FdhAB and AldB-II overtook detoxification at high aldehyde concentrations. Deletion strains were constructed and characterized towards unveiling these biochemical mechanisms, underscoring the worth of *P. putida* for emergent biotechnological applications—e.g. engineering synthetic formatotrophy and methylotrophy.

**IMPORTANCE:**

C1 substrates continue to attract interest in biotechnology, as their use is both cost-effective and ultimately expected to mitigate the impact of greenhouse gas emissions. However, our current understanding of bacterial C1 metabolism remains relatively limited in species that cannot grow on (i.e., assimilate) these substrates. *Pseudomonas putida*, a model Gram-negative environmental bacterium, constitutes a prime example of this sort. The biochemical pathways active in response to methanol, formaldehyde, and formate have been largely overlooked—although the ability of *P. putida* to process C1 molecules has been previously alluded to in the literature. By using a systems-level strategy, this study bridges such knowledge gap through the identification and characterization of mechanisms underlying methanol, formaldehyde, and formate detoxification—including hitherto unknown enzymes that act on these substrates. The results reported herein both expand our understanding of microbial metabolism and lay a solid foundation for engineering efforts toward valorizing C1 feedstocks.

## INTRODUCTION

*Pseudomonas putida* is a soil bacterium and plant-root colonizer endowed with high tolerance toward multiple toxic molecules, paired with a versatile metabolism that enables rapid adaption to changing environments (1). When grown on sugars, *P. putida* relies on the EDEMP cycle [a combination of enzymes from the Entner-Doudoroff (ED), the pentose phosphate (PP), and the (incomplete) Embden-Meyerhof-Parnas (EMP) pathways] to favor catabolic NADPH formation, which is not only essential for anabolic functions but also needed to cope with oxidative stress (2, 3). *P. putida* is able to metabolize several other carbon sources, including aromatic hydrocarbons and lignocellulosic-derived feedstocks (4 - 7). Yet, one-carbon (C1) feedstocks are not part of the (remarkably broad) range of substrates processed by *P. putida* through its native metabolism (8). C1 substrates continue to gain interest toward replacing sugars as substrates for bioproduction (9, 10), as they can be efficiently produced from CO_2_ *via* electrochemical reaction (11)—contributing to reduce greenhouse gas emissions by valorizing CO_2_ into both commodity and specialty products.

Owing to its advantageous metabolic and physiological properties, *P. putida* has been extensively engineered for the biosynthesis of a number of value-added chemicals—e.g., rhamnolipids (12), polyhydroxyalkanoates (13), anthranilate (14), *cis*,*cis*-muconate (15), and more recently, fluorinated building blocks (16 - 18). These engineering efforts were aided by the ongoing development of a broad set of tools that enable efficient genetic modifications (19). Such protocols also facilitated the construction of reduced-genome strains derived from *P. putida* KT2440. *P. putida* EM42, for instance, exhibits a higher biomass yield and increased production of heterologous proteins as compared to its parental strain (20), making it an attractive bioproduction host. In addition, genome sequencing and careful annotation (21, 22) support efficient metabolic rewiring in this species (23). However, functional validation is still missing for a number of genes [1,151 genes are still linked to unknown functions/hypothetical proteins (24)] toward fully understanding and harnessing the biotechnological potential of *P. putida*. Fatty acid and alcohol metabolism is a prime example of this sort. A recent study identified key players involved in the catabolism of various such compounds at the genetic level (25), but the mechanistic and regulatory aspects of the cognate biochemical steps remain largely unknown. Along the same lines, metabolic mechanisms for methanol, formaldehyde, and formate dissimilation and detoxification in *P. putida* have not been investigated at a systems level. Both NAD^+^- and pyrroloquinoline quinone (PQQ)-dependent alcohol dehydrogenases, displaying broad substrate specificity, are known to be involved in the initial oxidative processing of some alcohols and aldehydes (25, 26). AdhP, PedE, and PedH, for instance, have been shown to oxidize methanol (27), while *yiaY* overexpression enhanced methanol dissimilation in engineered *P. putida* strains (28). Together with these biochemical activities, the natural resistance of *P. putida* toward solvents and alcohols highlights its potential to host synthetic methanol assimilation pathways (29, 30). Additionally, the genome of *P. putida* encodes multiple NAD^+^-dependent formaldehyde (i.e., *fdhA* and *fdhB*) and formate dehydrogenases (FDHs, e.g., *fmdEFGH*)—enabling the use of these C1 molecules as co-substrates to boost ATP formation (31 - 33). Again, a deep understanding of the genetic and molecular basis of the native methanol, formaldehyde, and formate oxidation capabilities of *P. putida* is required—especially toward informing metabolic engineering of C1 assimilation.

On this background, we set out to identify native dissimilation and detoxification mechanisms in *P. putida* by applying a genome-wide RNA sequencing (RNA-Seq) approach. Upon investigating differentially expressed genes in the presence of methanol and formate, quantitative physiology experiments with both the wild-type strain and a combinatorial set of single- and multiple-deletion mutants were implemented to functionally validate the results of the transcriptional analysis. This experimental approach allowed for the identification of two novel formate dehydrogenases encoded in the genome of *P. putida*. Furthermore, we demonstrate that in the active oxidation of methanol to formaldehyde (and, to some extent, formate) underlies the apparent sensitivity phenotype of *P. putida* when exposed to the C1 alcohol. The results presented in this work underscore an updated model for the metabolic mechanisms involved in the dissimilation and detoxification of C1 molecules by *P. putida*—paving the way for engineering efforts toward biotechnological valorization of these substrates.

## MATERIALS AND METHODS

### Chemicals and reagents

Chemicals were purchased from Sigma-Aldrich Co. (St. Louis, MO, USA) unless otherwise indicated, and oligonucleotides were synthesized by Integrated DNA Technologies Inc. (Coralville, IA, USA). DNA sequencing was performed at Eurofins Genomics (Ebersberg, Germany). All primers used in this study are listed in Table S3. PCR reactions were performed using Phusion *U* Hot Start^TM^ DNA polymerase, purchased from ThermoFisher Scientific Co. (Waltham, MA, USA). The commercial One*Taq* ^TM^ master mix from New England BioLabs (Ipswich, MA, USA) was used for colony PCRs.

### Bacterial strains, medium composition and culture conditions

All bacterial strains and plasmids are listed in Table 1 and Table 2, respectively. *E. coli* DH5α λ*pir* was used as a cloning host, while the reduced-genome *P. putida* strain EM42 (21) was selected for quantitative physiology and engineering purposes. Lysogeny broth (LB) complex medium (containing 10  g L^−1^ tryptone, 5  g L^−1^ yeast extract, and 10  g L^−1^ NaCl) and de Bont minimal (DBM) medium were used for all cultivations (34). DBM medium contained 3.88 g L^−1^ K_2_HPO_4_, 1.63 g L^−1^ NaH_2_PO_4_, 2 g L^−1^ (NH_4_)_2_SO_4,_ and 0.1 g L^−1^ MgCl_2_∙6H_2_O, with the initial pH adjusted at 7.0. DBM medium was supplemented with a trace element solution [10 mg L^−1^ ethylenediaminetetraacetic acid (EDTA), 2 mg L^−1^ ZnSO_4_∙7H_2_O, 1 mg L^−1^ CaCl_2_∙2H_2_O, 5 mg L^−1^ FeSO_4_∙7H_2_O, 0.2 mg L^−1^ Na_2_MoO_4_∙2H_2_O, 0.2 mg L^−1^ CuSO_4_∙5H_2_O, 0.4 mg L^−1^ CoCl_2_∙6H_2_O, and 1 mg L^−1^ MnCl_2_∙2H_2_O]. When needed, kanamycin (Km) and gentamicin (Gm) were supplied at 50 µg mL^−1^ and 10 µg mL^−1^, respectively. All other cultivations were carried out in the absence of antibiotic selection.

**TABLE 1:** Bacterial strains used in this study

Strain	Relevant characteristics^*a*^	Reference or source
*Escherichia coli*		
DH5α λ*pir*	Cloning host; F^–^ λ*^–^ endA1 glnX44(AS) thiE1 recA1 relA1 spoT1* *gyrA96*(Nal^R^) *rfbC1 deoR nupG* F*80(lacZ*∆*M15)* ∆*(argF-lac)U169* *hsdR17(r_K_^–^ m_K_^+^)*, λ*pir* lysogen	Platt et al. (35)
*Pseudomonas putida*		
EM42	Reduced-genome derivative of *P. putida* KT2440 (24); ∆*PP_4329-PP_4397* (flagellar operon) ∆*PP_3849-PP_3920* (prophage I) ∆*PP_3026-PP_3066* (prophage II) ∆*PP_2266-* *PP_2297* (prophage III) ∆*PP_1532-PP_1586* (prophage IV) ∆*Tn7* ∆*endA-1* ∆*endA-2* ∆*hsdRMS* ∆Tn*4652*	Martínez-García et al. (21)
∆*FDH1*	Derivative of *P. putida* EM42, ∆*fdoGHI*-*fdhE*	Turlin et al. (36)
∆*FDH2*	Derivative of *P. putida* EM42, ∆*fmdEFGH*	Turlin et al. (36)
∆∆*FDH*	Derivative of *P. putida* EM42, ∆*fdoGHI*-*fdhE* ∆*fmdEFGH*	Turlin et al. (36)
∆*PP_0256-0257*	Derivative of *P. putida* EM42, ∆*PP_0256-57*	This work
∆*PP_4596*	Derivative of *P. putida* EM42, ∆*PP_4596*	This work
∆∆∆∆*FDH*	Derivative of *P. putida* EM42, ∆*fdoGHI*-*fdhE* ∆*fmdEFGH* ∆*PP_0256-57* ∆*PP_4596*	This work
∆*frmAC*	Derivative of *P. putida* EM42, ∆*frmAC*	This work
∆*aldB-II*	Derivative of *P. putida* EM42, ∆*aldB-II*	This work
∆*fdhA*	Derivative of *P. putida* EM42, ∆*fdhA*	This work
∆*fdhB*	Derivative of *P. putida* EM42, ∆*fdhB*	This work
∆*frmAC* ∆*aldB-II* ∆∆*fdhAB*	Derivative of *P. putida* EM42, ∆*frmAC* ∆*aldB-II* ∆*fdhA* ∆*fdhB*	This work
∆*yiaY*	Derivative of *P. putida* EM42, ∆*yiaY*	This work
∆*pedE*	Derivative of *P. putida* EM42, ∆*pedE*	This work
∆*pedH*	Derivative of *P. putida* EM42, ∆*pedH*	This work
∆*adhP*	Derivative of *P. putida* EM42, ∆*adhP*	This work
∆*yiaY* ∆∆*pedEH* ∆*adhP*	Derivative of *P. putida* EM42, ∆*yiaY* ∆*pedE* ∆*pedH* ∆*adhP*	This work

^*a*^ Antibiotic markers: Nal, nalidixic acid.

**TABLE 2:** Plasmids used in this study

Plasmid	Relevant characteristics^*a*^	Reference or source
pGNW2	Suicide vector used for deletions in Gram-negative bacteria; *oriT*, *traJ*, *lacZ*α, conditional RK6 replication origin, P_EM7_→*msfGFP*; Km^R^	Wirth et al. (37)
pGNW2·∆*fmdEFGH*	Suicide vector derivative of pGNW2 for deleting *fmdEFGH*; Km^R^	Turlin et al. (36)
pGNW2·∆*fdoGHI-fdhE*	Suicide vector derivative of pGNW2 for deleting *fdoGHI-fdhE*; Km^R^	Turlin et al. (36)
pGNW2·∆*PP_0256-57*	Suicide vector derivative of pGNW2 for deleting *PP_0256-57*; Km^R^	Turlin et al. (36)
pGNW2·∆*PP_4596*	Suicide vector derivative of pGNW2 for deleting *PP_4596*; Km^R^	This work
pGNW2·∆*frmAC*	Suicide vector derivative of pGNW2 for deleting *frmAC*; Km^R^	This work
pGNW2·∆*aldB-II*	Suicide vector derivative of pGNW2 for deleting *aldB-II*; Km^R^	This work
pGNW2·∆*fdhA*	Suicide vector derivative of pGNW2 for deleting *fdhA*; Km^R^	This work
pGNW2·∆*fdhB*	Suicide vector derivative of pGNW2 for deleting *fdhB*; Km^R^	This work
pGNW2·∆*yiaY*	Suicide vector derivative of pGNW2 for deleting *yiaY*; Km^R^	This work
pGNW2·∆*pedE*	Suicide vector derivative of pGNW2 for deleting *pedE*; Km^R^	This work
pGNW2·∆*pedH*	Suicide vector derivative of pGNW2 for deleting *pedH*; Km^R^	This work
pGNW2·∆*adhP*	Suicide vector derivative of pGNW2 for deleting *adhP*; Km^R^	This work
pQURE6·H	Helper plasmid for gene deletions; conditionally replicating vector carrying XylS/*Pm*→*I-SceI* and P_14g_(BCD2)→*mRFP*; Gm^R^	Volke et al. (38)
pS621·*PP_0256-57*	Derivative of vector pSEVA621 (39) harboring a *P_trc_* promoter and the canonical SEVA ribosome binding site (RBS)→*PP_0256-57*; *oriT oriV*(RK2); Gm^R^	This work
pS621c	Control vector derivative of pSEVA621 (39) harboring a *P_trc_* promoter and the canonical SEVA ribosome binding site (RBS); *oriT oriV*(RK2); Gm^R^	This work

^*a*^ Antibiotic markers: Gm, gentamicin; Km, kanamycin.

Overnight cultures in LB medium were diluted 1/100 to inoculate a 5 mL preculture of DBM medium with 20 mM glucose in a 50mL culture tube and incubated at 30°C and 250 rpm for ca. 18 h. This overnight culture was washed with DBM medium without any carbon source prior to the inoculation of the main culture in either 96-well microtiter plates or baffled Erlenmeyer flasks (shaken-flask cultures) with the appropriate carbon source(s) as described in the text. For cultivations in 96-well microtiter plates, 200 µL of a cell suspension at an OD_600_ of 0.05 were incubated in an ELx808 microtiter plate reader (BioTek Instruments Inc., Winooski, VT, USA). Shaken-flask cultivations were performed in 50 mL of medium in 250 mL Erlenmeyer flasks at 30°C and 200 rpm. The specific growth rate (μ) and the extension of the lag phase (λ) were calculated by performing a smooth spline fit on the growth data (40).

### Construction of deletion plasmids

The suicide plasmids pGNW2·∆*PP_0256-57*, pGNW2·∆*PP_4596*, pGNW2·∆*frmAC*, pGNW2·∆*aldB-II*, pGNW2·∆*fdhA*, pGNW2·∆*fdhB*, pGNW2·∆*yiaY*, pGNW2·∆*pedE,* pGNW2·∆*adhP,* and pGNW2·∆*pedH* (Table 2) were constructed using USER cloning (41). DNA fragments, consisting of the 500 bp upstream and downstream regions around the locus to be eliminated, were amplified with Phusion *U* Hot Start DNA polymerase (ThermoFisher Scientific Co.) using uracil-containing primers. The pGNW2 backbone (37) was digested with *Dpn*I prior to mixing 1 µL of *Dpn*I-treated vector with 100 ng of each PCR fragment and 1 µL of USER^TM^ enzyme (New England BioLabs) in a final volume of 10 µL. The reaction was incubated for 30 min at 37°C, followed by a temperature decrease over 3 min (from 28°C to 20°C, 2°C per step) and a final incubation step at 10°C for at least 10 min. Finally, chemically competent *E. coli* DH5α λ*pir* cells (42) were mixed with 5 µL of the USER mix and transformed *via* heat shock; upon recovery, the cell suspension was plated onto selective LB medium agar plates containing the corresponding antibiotic.

### Construction of mutant *P*. *putida* strains

The corresponding suicide pGNW2-derivative plasmid was delivered into the cells by electroporating 500 ng of plasmid DNA into 50 µL of freshly prepared electrocompetent *P. putida* EM42 cells, previously washed three times with 300 mM sucrose. Electroporation was performed with a Gene Pulser XCell (Bio-Rad) set to 2.5 kV, 25 µF capacitance, and 200 Ω resistance in a 2 mm gap cuvette. Positive co-integration events were further transformed with pQURE6·H (Table 2), a conditionally replicative plasmid bearing the meganuclease gene I-*Sce*I. I-*Sce*I cuts pGNW2 co-integrants within the chromosome, thus forcing a second homologous recombination event. Cells were recovered in 1 mL of LB medium supplemented with 2 mM of 3-methylbenzoate (3-*m*Bz) for at least 3 h at 30°C and plated onto LB medium agar containing the corresponding antibiotic(s) and 1 mM 3-*m*Bz to induce both plasmid replication and I-*Sce*I expression. Positive clones were identified by colony PCR, verified by DNA sequencing, and cured from the resolving plasmid by serial dilution under nonselective conditions.

### Analytical procedures for quantitative physiology experiments

The concentration of glucose, formate, and methanol was measured in culture supernatants in a high-performance liquid chromatography (HPLC) instrument (UltiMate 3000 Basic Automated System ThermoFisher Scientific Co) equipped with an Aminex HPX-87P column (Bio-Rad) and a refractive index detector Shodex RI-101 (Showa Denko America Inc., NY, USA). Specific glucose (*q*_*G*_), formate (*q*_*F*_), and methanol (*q*_*M*_) consumption rates were normalized to the cell dry weight (CDW) with the following equation


$$q_{S}=\frac{1}{X}\frac{\Delta S}{\Delta t}$$

where *q*_*S*_ corresponds to the biomass-specific substrate consumption rate (mmol g_CDW_^−1^ h^−1^), *X* is the average biomass concentration between two sampling time points (g_CDW_ L^−1^), ∆*S* is the difference in substrate concentration between two sampling time points (mM), and ∆*t* refers to the time between two sampling points (h). The *q*_*G*,_ *q*_*F*,_ and *q*_*M*_ values correspond to an average of the values individually determined in three biological replicates.

### Comparative transcriptome analysis by RNA-Seq

Differential transcriptome analysis was used to explore gene expression in shaken-flask cultures of *P. putida* grown in the presence of formate or methanol. At the time(s) indicated in the text, an equivalent of 1 mL of culture at an OD_600_ of 1 was pelleted at 10,000×g for 10 min at 4°C, washed with phosphate-buffered saline and immediately frozen in liquid nitrogen. Pellets were stored at −80°C until further processed. RNA extraction, library preparation, and RNA-Seq were performed by BGI (Shenzhen, China) as indicated previously (43). Sequencing data were filtered by removing reads mapped to rRNA as well as sequencing reads containing low-quality, adaptor-polluted, and high content of unknown base reads. Next, clean reads were mapped to the reference bacterial genome (24) using HISAT2 (Hierarchical Indexing for Spliced Alignment of Transcripts) (44). The clean reads were mapped to reference transcripts using Bowtie2 (45), and the gene expression level for each sample was calculated with RSEM (46). RNA-Seq data were analyzed using *VisomX* version 0.0.0.9. Initially, missing values were filtered to remove genes that were not detected in every replicate of at least one condition. Furthermore, only genes with a count of ≥ 5 in at least three samples were retained. Differential expression analysis of log_2_-transformed read counts was performed with the function *DESeq* (with *sfType = “ratio*”) of the *DESeq2* package version 1.36.0 (47). *P*-values were adjusted by independent hypothesis weighting using the IHW package (48); log_2_(fold change) (FC) shrinkage was performed through the “apeglm” method of the *apeglm* package (49). Genes showing absolute log_2_(FC) values of ≥ 2 and adjusted *P*-values (*q*-values) of ≤ 0.01 were identified as differentially expressed.

### Statistical analysis

Data analysis was performed using MS Excel, Prism 9.0.2 (GraphPad Software Inc., San Diego, CA, USA) and *R* studio (Integrated Development for *R*; RStudio, PBC, Boston, MA, USA) unless differently specified. Reported values are indicated as averages ± standard deviation of replicates as specified in the legend of the corresponding figures. The level of statistical significance of differences when comparing results across experiments and conditions was evaluated by ANOVA (Dunnett’s test, Prism 9.0.2).

## RESULTS

### Native mechanisms of formate oxidation and tolerance in *P*. *putida* and other Gram-negative bacteria

Formic acid is a weak acid (pK*_a_* = 3.74), and its toxicity is associated with the diffusion across the cell membrane at low pH, thus acidifying the cytoplasm and impairing the proton motive force (50). Formate can also lead to oxidative stress and inhibit the activity of bacterial cytochrome *c* oxidases (51, 52). To overcome the toxicity brought about by this C1 acid, microorganisms have evolved different dissimilation mechanisms. The growth of microbes with weak FDH activity, e.g., *E. coli*, is impaired at formate concentrations below 100 mM (53). Bacteria bearing highly active FDHs, in contrast, can tolerate several hundred mM formate (54)*.*

**Fig 1:**
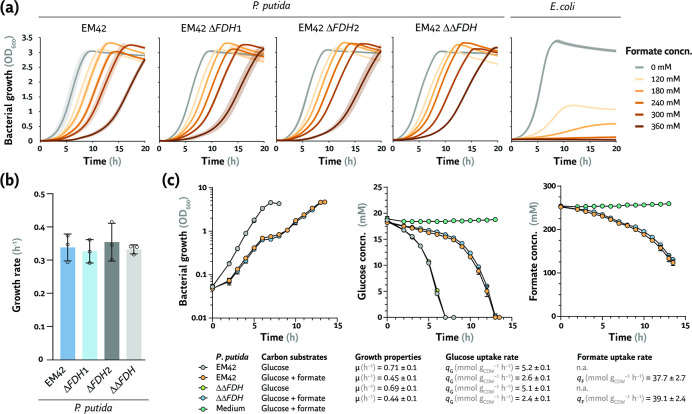
Assessing formate tolerance and native oxidation mechanisms in *E. coli* and *P. putida*. (a) Growth profiles of *P. putida* EM42 and *P. putida* harboring deletions in *fdoGHI-fdhE* (strain ∆*FDH*1), *fmdEFGH* (strain ∆*FDH*2), and the combined knockout of *fdoGHI-fdhE* and *fmdEFGH* (strain ∆∆*FDH*) in minimal medium supplemented with 20 mM glucose and increasing formate concentrations (concn.). *Escherichia coli* MG1655, included here as a control, was cultivated at 37°C in M9 minimal medium; *P. putida* was cultivated at 30°C in de Bont minimal medium. Average values for the bacterial growth (estimated as the optical density measured at 600 nm, OD_600_) ± standard deviation of three biological replicates are represented. (b) Growth rates of *P. putida* EM42 and its ∆*FDH*1, ∆*FDH*2, and ∆∆*FDH* derivatives cultivated in de Bont minimal medium with 20 mM glucose and 360 mM formate. Average values for the specific growth rate (μ) ± standard deviation of three biological replicates are represented; individual data points are included in the plots. (c) Quantitative physiology parameters for *P. putida* EM42 and the full ∆∆*FDH* mutant grown in shaken-flask cultures. Cultivations were performed in biological triplicates in de Bont minimal medium supplemented with 20 mM glucose and, when indicated, 240 mM formate. Growth and specific consumption rates were calculated during exponential growth; a non-inoculated culture (labeled as “Medium”) was included as a control. Average values ± standard deviation for the specific consumption rate of glucose (*q*_G_) and formate (*q*_F_), as well as specific growth rates, are indicated in the figure. *CDW*, cell dry weight; n.a., not applicable.

In a previous study, we showed that *P. putida* could tolerate formate at least at 240 mM in LB medium—suggesting high levels of endogenous FDH activity (36). *P. putida* possesses two *bona fide* FDHs (31, 32), i.e., FdoGHI-FdhE (PP_0489-PP_0492) and FmdEFGH (PP_2183-PP_2186). The *fdoGHI-fdhE* cluster encodes a membrane-bound FDH, possibly using quinol as a cofactor, while FmdEFGH is annotated to be a soluble, NAD^+^-dependent FDH. Both FDHs are predicted to contain iron-sulfur clusters and molybdenum-binding domains. Metal-containing FDHs exhibit enhanced kinetic properties toward formate oxidation than similar dehydrogenases, and they can also catalyze the reduction of CO_2_ to formate (55, 56). *In vitro* enzymatic assays highlighted that *P. putida* KT2440 metabolizes formate mostly through the soluble, NAD^+^-dependent FDH (31). In order to identify the role and contribution of the different FDHs of *P. putida* EM42 toward formate oxidation, we constructed several mutants where *fdoGHI-fdhE* (strain ∆*FDH*1) and *fmdEFGH* (strain ∆*FDH*2) were deleted, either independently or jointly (strain ∆∆*FDH*; Table 1). The growth profile of the resulting mutants was analyzed in DBM medium in the presence of 20 mM glucose as the main carbon source and increasing formate concentrations (Fig. 1a). In the absence of formate, *P. putida* EM42 grew with a specific growth rate (μ) of 0.75 h^–1^. A protracted lag phase and reduced μ were observed with rising formate concentrations—but the final OD_600_ was not severely affected. The μ of wild-type strain EM42 decreased to 0.35 h^–1^ in cultures with 360 mM formate (Fig. 1b). A similar behavior was observed for the single and double FDH mutants, displaying a μ of ca. 0.37 h^–1^ with 360 mM formate (Fig. 1b). Since a reduced formate tolerance was expected upon deleting dissimilatory FDHs, we wondered whether FdoGHI-FdhE and FmdEFG are active and involved in formate oxidation under the conditions tested. *E. coli* cultures were included as a control in these experiments. We previously reported on the growth defects when *E. coli* was grown in rich LB medium supplemented with >120 mM formate (36). The same qualitative pattern was observed here when *E. coli* MG1655 was incubated in M9 minimal medium supplemented with 20 mM glucose and formate (Fig. 1). Bacterial growth was already impacted by formate at 120 mM, decreasing the final OD_600_ by ca. two-thirds. Likewise, no growth was observed in the presence of formate concentrations >180 mM (Fig. 1a).

**Fig 2:**
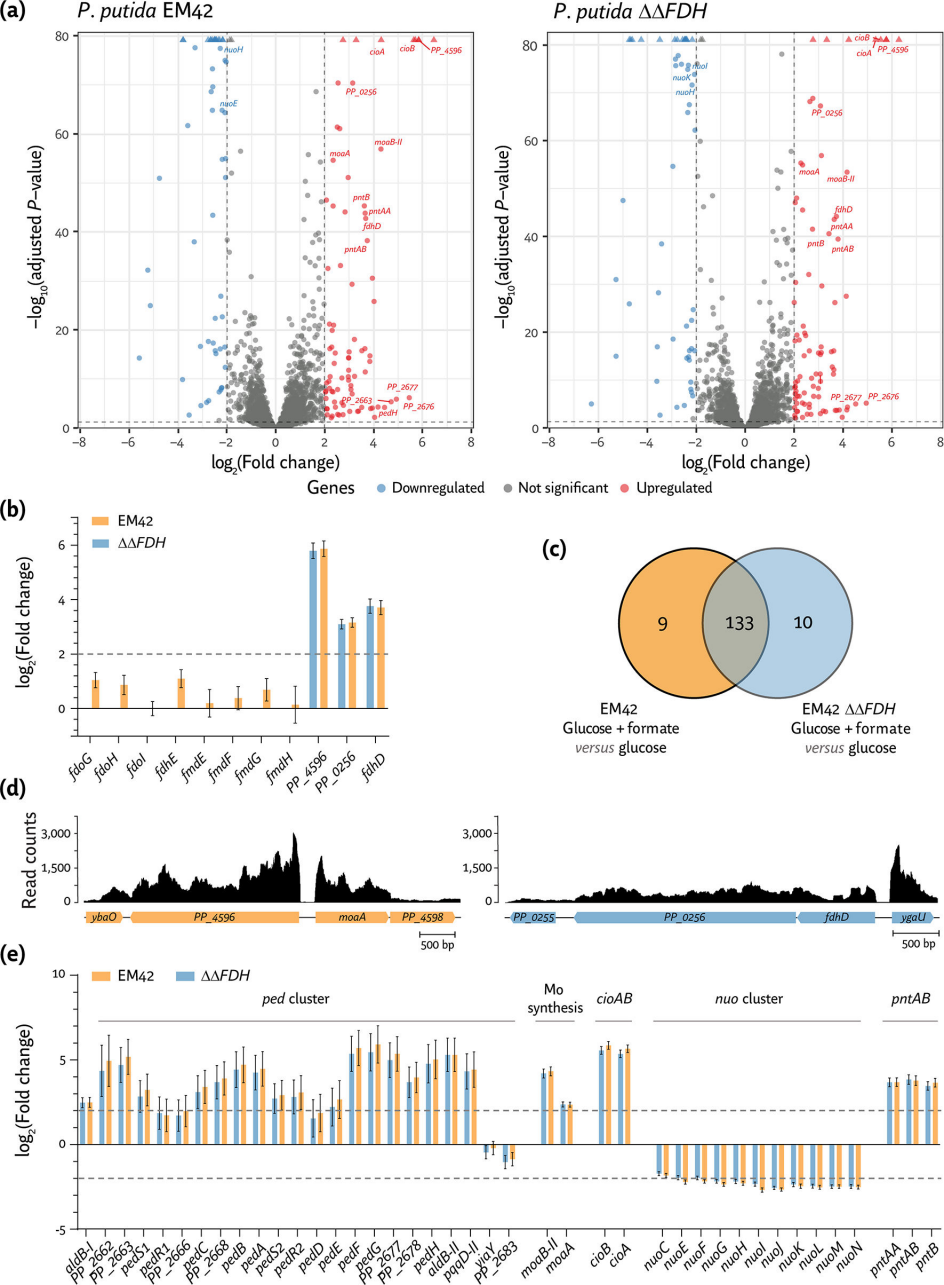
Differentially expressed genes in *P*. *putida* EM42 and the ∆∆*FDH* mutant cultivated in the presence of formate. Volcano plots showing differentially expressed genes (DEGs) in (a) *P. putida* EM42 or *P. putida* ∆∆*FDH* (∆*fdoGHI-fdhE* ∆*fmdEFGH*) grown in de Bont minimal medium supplemented with 20 mM glucose and 240 mM formate compared to *P. putida* EM42 cultivated with 20 mM glucose. Cells were harvested in the mid-exponential phase to identify DEGs. Each point in the volcano plot represents a single gene; the horizontal intersection was set at a *q*-value [FDR, false discovery rate, calculated as –log_10_(adjusted *P*-value)] ≤ 0.01 and the vertical intersections at a |log_2_(fold change)| ≥ 2. (b) Log_2_(Fold change) of a subset of significantly DEGs encoding functions related to formate oxidation. (c) Venn diagram of all DEGs in cells cultivated in the presence of glucose and formate as compared to *P. putida* EM42 grown on glucose only. The diagram highlights the overlap for each pairwise comparison. (d) Sequence coverage plot of a genome segment surrounding the *PP_4596* and *PP_0256-PP_0257* gene clusters. (e) Log_2_(fold change) of a subset of significant in *P. putida* EM42 or *P. putida* ∆∆*FDH* grown in de Bont minimal medium supplemented with 20 mM glucose and 240 mM formate compared to *P. putida* EM42 cultivated with 20 mM glucose.

Next, *P. putida* EM42 and its double ∆∆*FDH* mutant derivative were cultivated in DBM medium with 20 mM glucose in shaken-flask cultures, with or without 240 mM formate, and changes in the substrates concentration were measured over time (Fig. 2c). Glucose consumption mirrored bacterial growth, with a specific glucose uptake rate (*q*_G_) of 5.2 ± 0.1 mmol g of cell dry weight (CDW)^–1^ h^–1^ in the absence of formate and ca. 2.6 ± 0.1 mmol g_CDW_^–1^ h^–1^ in cultures added with formate (Fig. 2c). Bacterial growth slowed down upon formate supplementation (at 6 h of cultivation, corresponding to an OD_600_ of ca. 0.6–0.7) and resumed only after 2 h (Fig. 2c). This observation indicates that a sudden formate challenge results in an acute toxicity effect that stalls bacterial growth while detoxification mechanisms are being deployed. Feeding formate to cultures of *P. putida* KT2440 as an additional energy source has been shown to increase biomass yields (*Y*_X/S_) (31). A similar behavior was observed in this study, with an increase in *Y*_X/S_ of ca. 8.5% (on a glucose basis) upon supplementing formate to cultures of *P. putida* EM42. Glucose and formate were co-consumed (Fig. 2c). Surprisingly, the pattern and rate of formate uptake (*q*_F_) were similar in all cultures (with *q*_F_ = 37.7 ± 2.7 and 39.1 ± 2.4 mmol g_CDW_^–1^ h^–1^ for the wild-type EM42 strain and the ∆∆*FDH* mutant, respectively). With these results at hand and considering that no differences in formate tolerance or oxidation were observed upon deleting *fdoGHI-fdhE* and/or *fmdEFGH*, we explored the possibility that additional dehydrogenases might be present in *P. putida*. Hence, we set to study its genome-wide transcriptome to identify putative FDH candidates as explained below.

### High-resolution RNA sequencing reveals novel routes of formate oxidation

Shaken-flask cultures of *P. putida* EM42 and *P. putida* ∆∆*FDH* (in DBM medium with 20 mM glucose and with or without 240 mM formate) were harvested in mid-exponential phase (corresponding to 3 h for the glucose-only condition and 4 h for cultures added with formate) and processed for RNA-Seq analysis. A concentration of 240 mM formate was selected for these experiments as *E. coli* was not able to grow under this condition, while *P. putida* had a noticeable growth defect (Fig. 1a). Thus, we hypothesized that the effects exerted by formate toxicity on gene regulation would be evidenced in this culture setup. Changes in transcriptional levels could be reproducibly quantified for 5,158 genes of *P. putida* (Fig. 2a–c). Principal component analysis (PCA) of the total transcriptome data underscored high reproducibility across biological triplicate samples and illustrated different clustering patterns depending on medium composition (Fig. S1).

When comparing the global transcriptome of *P. putida* EM42 grown on glucose *versus* glucose cultures supplemented with formate, 142 DEGs could be identified—while 143 DEGs were recognized when the transcriptome of *P. putida* ∆∆*FDH* growing in the presence of formate and glucose was compared to the control, glucose-only experiment (Fig. 2c). Cultivating either the wild-type or the ∆∆*FDH* mutant in the presence of formate triggered transcriptional changes in 133 genes that were shared by both *P. putida* strains. Then, we compared the fold change (FC) of these DEGs to the transcriptional levels observed in the wild type growing on glucose and grouped them into genes (i) upregulated or (ii) downregulated in the presence of formate (Fig. 2a; Table S1). As expected, a major transcriptional difference between *P. putida* EM42 and its ∆∆*FDH* derivative grown in the presence of formate lies in the expression of *fdoGHI-fdhE* and *fmdEFGH* (Fig. 2b)—as these genes were deleted in the double mutant strain (Table 1). The majority of the other open reading frames (ORFs) were differentially expressed to a similar extent as compared to *P. putida* EM42 cultivated with glucose (Fig. 2a–c; Table S2). In fact, and as indicated above, ca. 93% of the identified DEGs overlapped between the two strains—highlighting a strong similarity in the transcriptional response of *P. putida* to formate challenges, regardless of the presence of the two (supposedly major) FDHs (Fig. 2c; Fig. S1). Quite surprisingly, neither *fdoGHI-fdhE* nor *fmdEFGH* was significantly upregulated when *P. putida* EM42 was cultivated in the presence of 240 mM formate (Fig. 2b). This observation could be indicative of either (i) high levels of constitutive expression for the *fdo* and *fmd* genes, (ii) post-transcriptional regulation of the cognate proteins, or (iii) a relatively minor role of these FDHs in formate processing. This result prompts the question of whether other dehydrogenases are involved in formate dissimilation.

*PP_4596* and *PP_0256*, annotated to encode two soluble oxidoreductases that belong to the broad FdhF/YdeP family, were significantly overexpressed in both *P. putida* EM42 and strain ∆∆*FDH* upon exposure to the C1 acid (Fig. 2a). FdhF/YdeP oxidoreductases usually contain a molybdopterin cofactor and are associated with other dehydrogenase subunits featuring iron-sulfur clusters (57). The polypeptides encoded by *PP_4596* and *PP_0256* share little similarity to known FDHs of *P. putida*, *Methylobacterium extorquens*, *E. coli,* or *Cupriavidus necator* [20 to 35% identity, according to BLAST analysis (58)]. However, PP_4596 and PP_0256 display a significant degree of amino acid identity with Fdh4A from *M. extorquens* (59%; Fig. S2) and the YdeP protein of *E. coli* (47% and 49% for PP_4596 and PP_0256, respectively; Fig. S3). Fdh4A was demonstrated to be involved in formate oxidation (59), whereas YdeP was shown to be connected to acid stress responses (60). Interestingly, PP_4596 and PP_0256 also exhibit 1% amino acid identity to each other (Fig. S4), hinting at similar functionality of the polypeptides. The gene encoding FdhD (*PP_0257*), a FDH accessory sulfurtransferase, was also highly overexpressed in the presence of formate (33). The sequence coverage plot of these loci at the single-nucleotide resolution suggests that *PP_4596* constitutes a separate, single transcriptional unit, whereas *fdhD* and *PP_0256* are part of the same mRNA transcript (Fig. 2d). FdhD is a carrier protein that transfers sulfur to the molybdenum cofactor prior to its insertion into formate dehydrogenases. FdhD activity is induced upon cultivation of *P. putida* in the presence of sublethal formaldehyde concentrations (61). Taken together, the results described thus far suggest that FdoGHI-FdhE and FmdEFGH are not the primary FDHs; the experiments also indicate that the PP_4596 and PP_0256 oxidoreductases are actively involved in formate oxidation in *P. putida*.

### Exposure of *P*. *putida* to formate triggers network-wide redox balancing mechanisms

In addition to genes encoding putative FDHs, *moaA* and *moaB-II*, which encode the cyclic pyranopterin monophosphate synthase (PP_4597) and the molybdenum cofactor biosynthesis protein B (PP_4600), were highly expressed in the presence of formate [log_2_(FC) = 2.4 and 4.3 for *moaA* and *moaB-II*, respectively; Fig. 2e]. Overexpression of genes within the molybdopterin biosynthesis pathway positively correlates with the activation of molybdenum-dependent FDHs. This transcriptional pattern was shared with several genes of the *ped* cluster that encodes PQQ-dependent alcohol dehydrogenases (Fig. 2e), known to be active when *P. putida* is cultivated in the presence of alcohols (25). PedE and PedH act on an extensive range of alcohols and aldehydes with a strong dependence on PedF, a cytochrome *c* oxidase that regenerates the PQQ cofactor (27). All of these genes were overexpressed in strain EM42 cultivated in the presence of formate [log_2_(FC) = 2.6 for *pedE*, 5.1 for *pedH,* and 5.7 for *pedF*; Fig. 2e].

Genes encoding respiratory components were also differentially regulated in the presence of formate. *P. putida* harbors five terminal oxidases, i.e., Cio, Cyo (a *bo3*-type oxidase), and the cytochromes *aa3*, *cbb3-1,* and *cbb3-2* oxidases (24). These terminal oxidases display differential redox potentials, regulatory patterns, O_2_ affinity, and H^+^ pumping efficiency (62). The *cioA* and *cioB* genes, encoding the cyanide-insensitive ubiquinol oxidase subunits I and II (PP_4651 and PP_4650), and members of the cytochrome *bd* family were among the most significantly differentially expressed (Fig. 2a and e). Cio receives electrons from the ubiquinol pool in the periplasmic space and is involved in oxidative and nitrosative stress defense (63). In *E. coli*, cytochrome *bd* expression and content increase at low O_2_ availability (64), medium alkalization (65), high temperature (66), and in the presence of cyanide (67). Hence, the pH increase triggered by formate addition could correlate with *cioAB* overexpression in *P. putida*. Conversely, the expression of genes encoding NuoABCEFGHIJKLMN, the NADH-quinone oxidoreductase component of the electron transport chain (complex I; PP_4119-PP_4131), was significantly downregulated in the presence of formate [log_2_(FC) < –2; Fig. 2e]. Fine-tuning adjustments of the respiratory chain activity in response to the C1 acid remain to be fully elucidated—but these observations point to a differential pattern of redox balancing. Changes in the expression of genes involved in intracellular redox homeostasis support this notion. Indeed, the membrane-bound, proton-pumping pyridine nucleotide transhydrogenase genes (*pntAA*, *PP_0156*, *pntAB*, *PP_5747* and *pntB*, *PP_0155*) were overexpressed in formate-challenged *P. putida* cells (Fig. 2e). In *E. coli*, PntAB catalyzes the reduction of NADP^+^ by NADH, regenerating NADPH while transferring H^+^ inside the cell (68). While NADH is linked to ATP generation *via* oxidative phosphorylation, NADPH is mainly used as a cofactor for anabolism and as a reductant in oxidative stress responses (69). Our results indicate that formate addition activates the expression of NAD^+^-dependent FDHs in *P. putida*, thereby affecting redox homeostasis; *pntAB* overexpression hints at redox balancing mechanisms that ultimately yield NADPH *via* transhydrogenation (70).

### Functional validation of PP_04596 and PP_0256 as major FDHs of *P*. *putida*

**Fig 3:**
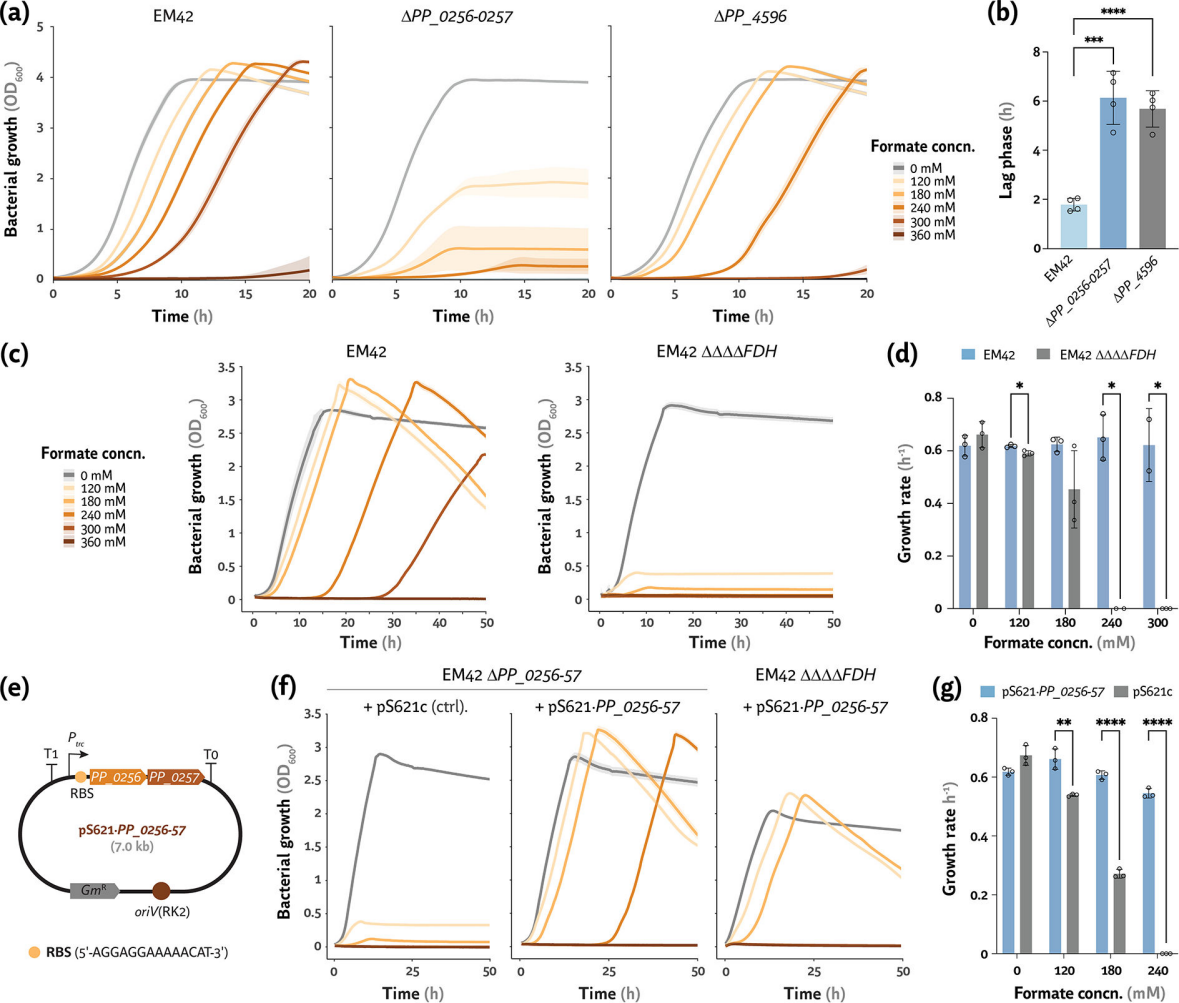
Identification of two formate dehydrogenases in *P*. *putida* and functional validation of their role in formate tolerance. (a) Formate tolerance of *P. putida* EM42 and its mutant derivative harboring deletions of *PP_0256-PP_0257* or *PP_4596*, encoding putative formate dehydrogenases, in cultures supplemented with increasing formate concentrations (concn.). (b) The extension of the lag phase (λ) was calculated for cultures of *P. putida* EM42, ∆*PP_0256-0257,* and ∆*PP_4596* cultivated in the presence of 240 mM formate. (c and d) Growth patterns of a quadruple mutant of *P. putida* EM42 depleted of all four formate dehydrogenases (strain ∆∆∆∆*FDH*, ∆*fdoGHI-fdhE* ∆*fmdEFGH* ∆*PP_0256-57* ∆*PP_4596*) against the parental strain. (e-g) Complementation assays with plasmid pS621·*PP_0256-57*, expressing the *PP_0256-57* operon under transcriptional control of the constitutive *P_trc_* promoter; the empty pS621c vector was used in control (ctrl.) experiments. The ∆∆∆∆*FDH* strain transformed with the pS621c vector had similar growth profiles as *P. putida* ∆*PP_0256-57* ∆*PP_4596*/pS621c at all formate concn. *RBS*, ribosome binding site; *Gm^R^*, gentamicin-resistance determinant. The color code used to identify different formate concn. is the same as for panels (a) and (c). All bacterial cultivations were performed in de Bont minimal medium containing 20 mM glucose. Average values for bacterial growth (estimated as the optical density measure at 600 nm, OD_600_), λ, and specific growth rate (μ) ± standard deviation of three biological replicates are represented in all cases. Individual data points are shown whenever relevant. The level of significance (according to the Dunnett’s test) is indicated with asterisks as follows: ∗ *P*-value < 0.05, ** *P*-value < 0.01 and ∗∗∗∗ *P*-value < 0.0001

The overexpression of genes encoding PP_4596, PP_0256, and FdhD in the presence of formate (Fig. 2a) suggested a role for these oxidoreductases in processing the C1 acid. To further investigate this possibility, the *PP_4596* and *PP_0256-fdhD* genes were independently deleted in *P. putida* EM42 to yield strains ∆*PP_4596* and ∆*PP_0256-0257*, respectively (Table 1). Next, the growth profile of these mutant strains was analyzed in DBM medium cultures supplemented with 20 mM glucose and increasing formate concentrations (Fig. 3A). The deletion of *PP_0256-0257* drastically affected formate tolerance, with growth deficiency starting at 120 mM and no growth observed above 240 mM formate (Fig. 3a). The deletion of *PP_4596*, on the other hand, had a milder effect on formate tolerance by *P. putida*—an increase in the lag phase was evident starting from 240 mM, and no growth was observed at >300 mM formate (Fig. 3a). Indeed, when cultivated with 240 mM formate, both mutant strains had a lag phase longer than 5.5 h (whereas the wild-type strain had a ca. 1.8 h lag time under the same conditions; Fig. 3b). The next step was combining all four deletions in a single mutant strain in order to explore the synergies of the FDHs in the formate tolerance phenotype of *P. putida*. To this end, strain ∆*fmdEFGH* ∆*fdoGHI-fdhE* ∆*PP_0256-0257* ∆*PP_4596* (termed EM42 ∆∆∆∆*FDH* for the sake of simplicity) was constructed (Table 1), and its growth phenotype was tested in DBM medium with different formate concentrations (Fig. 3c). As expected, eliminating all four FDHs of *P. putida* resulted in a severe effect in formate tolerance, rather similar to that observed for the single *PP_0256-0257* deletion (Fig. 3a). Indeed, the quadruple mutant could barely grow in the presence of 120 mM formate, and no growth was observed at >180 mM (unlike the parental strain EM42)—as reflected in the specific growth rates calculated under these conditions (Fig. 3d).

Since a similar growth defect was observed for *P. putida* ∆*PP_0256-0257* and ∆∆∆∆*FDH* when exposed to the C1 acid, complementation assays were set by cloning *PP_0256* and its neighboring *PP_0257* (*fdhD*) gene as a single transcriptional unit in plasmid pS621·*PP_0256-57* (Fig. 3e). In this plasmid, both genes are placed under the control of the constitutive *P_trc_* promoter (Table 2). Either the empty vector (p621c, used as a control) or plasmid pS621·*PP_0256-57* was introduced in different FDH-depleted strains, and the growth profiles of the resulting recombinants were analyzed in DBM medium supplemented with 20 mM glucose and increasing formate concentrations (Fig. 3f). Whereas *P. putida* ∆*PP_0256-57* carrying the empty p621c vector had a formate sensibility similar to that observed for the plasmid-less strain (Fig. 3c), plasmid pS621·*PP_0256-57* reverted the growth deficiency of the mutant virtually to the same levels observed for the wild-type strain (Fig. 3f). The μ values for strain EM42 and its ∆*PP_0256-57* derivative carrying plasmid pS621·*PP_0256-57* were practically the same in cultures containing ≤ 240 mM formate (Fig. 3g). Furthermore, the complementation effect was verified in strain ∆∆∆∆*FDH* (Fig. 3f), where expression of *PP_0256-57* restored growth in the presence of the C1 acid up to 240 mM. Overall, these results underscore the native ability of *P. putida* to withstand high formate concentrations and expose a hitherto unknown role for PP_4596 and PP_0256 as FDHs. We were also interested in exploring native mechanisms for processing the metabolites upstream formate, i.e., methanol and formaldehyde, as explained in the next section.

### Native methanol tolerance mechanisms in *P*. *putida* are linked to efficient routes for alcohol oxidation

**Fig 4:**
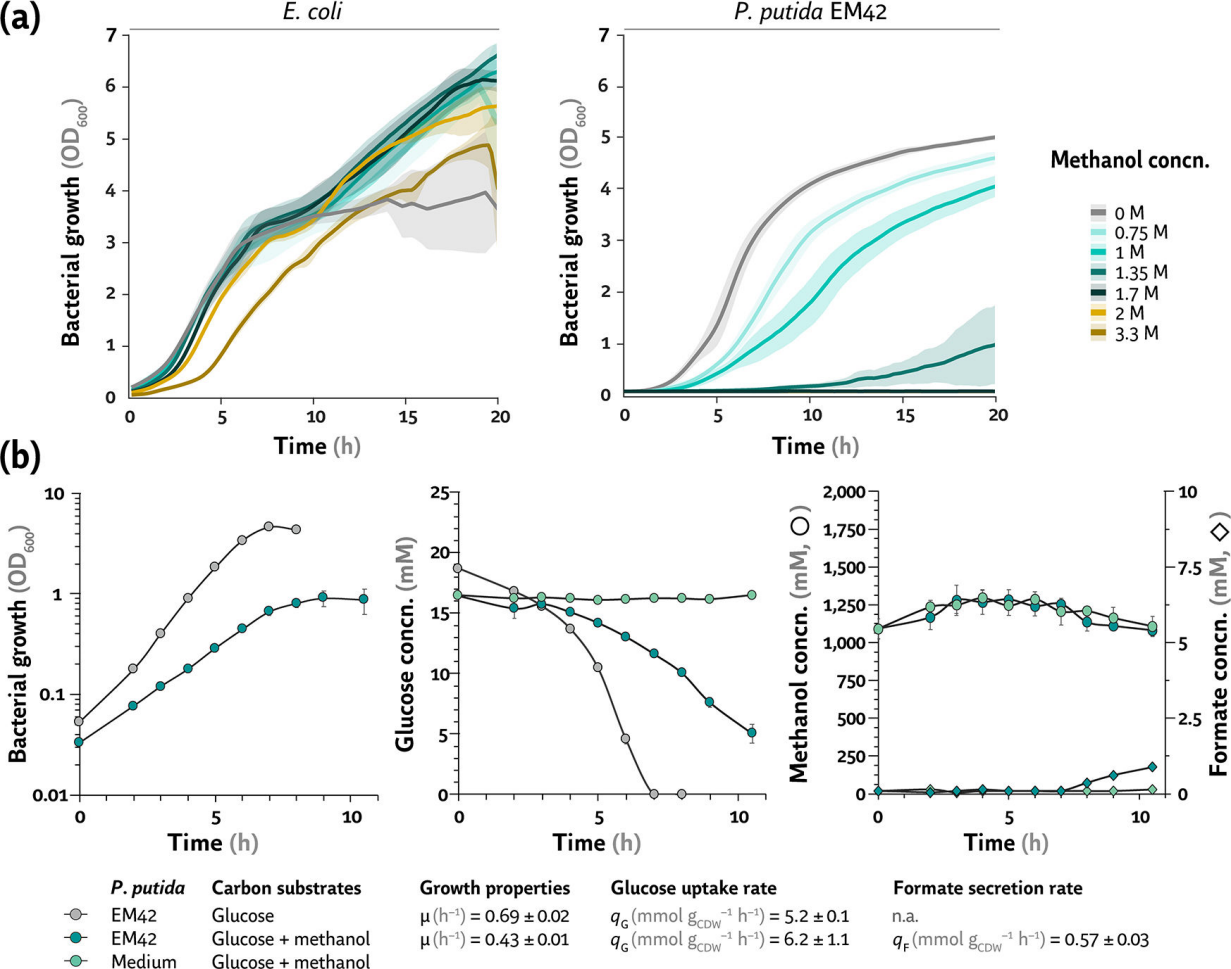
Assessing methanol tolerance and native routes of methanol metabolism in *E. coli* and *P. putida*. (a) Growth profiles of *E. coli* MG1655 and *P. putida* EM42 in minimal medium (either M9 or de Bont, respectively) supplemented with 20 mM glucose and increasing methanol concentrations (concn.). Average values for bacterial growth (estimated as the optical density measured at 600 nm, OD_600_) ± standard deviation of three biological replicates are plotted. (b) Growth profile of *P. putida* EM42 in shaken-flask cultures and patterns of glucose utilization, methanol oxidation, and formate secretion. Cultivations for quantitative physiology analysis were performed in biological triplicates in de Bont minimal medium supplemented with 20 mM glucose and, when indicated, 1 M methanol; a non-inoculated culture (labeled as “Medium”) was included in the analysis as control. Average values ± standard deviation for the specific consumption rate of glucose (*q*_G_) and secretion rate of formate (*q*_F_), as well as specific growth rates, were calculated during exponential growth and are indicated in the figure. *CDW*, cell dry weight; n.a., not applicable.

Methanol, a sustainable, easy-to-produce and naturally abundant one-carbon compound, has been studied as a substrate for biotechnology (71). Yet, methanol is a bacterial cytotoxic (72), and this effect is tied to either (i) alcohol intercalation between fatty acids, altering the fluidity and functioning of cell membranes (73, 74) or (ii) methanol oxidation to formaldehyde, which is highly reactive toward proteins and nucleic acids (75). Thus, microbial methanol tolerance comes from either limited alcohol oxidation or high dissimilatory formaldehyde activity to formate (and ultimately CO_2_). While *P. putida* KT2440 has been reported to tolerate up to 1.5 mM formaldehyde (61), no data on methanol tolerance is available. Hence, we determined the intrinsic methanol tolerance levels of *P. putida* EM42 in microtiter plate cultivations, using *E. coli* MG1655 as a control (Fig. 4). *E. coli* is known to grow in the presence of up to 10% (v/v) [ca. 2.5 M] methanol (76). Surprisingly, *P. putida* EM42 featured an apparently lower tolerance to methanol in our experiments when compared to *E. coli*. Strain EM42 tolerated methanol up to 1.35 M (although the final OD_600_ under these conditions was only one-fifth of that in control cultures), whereas *E. coli* was only affected by >3 M methanol (Fig. 4a). The *E. coli* growth profiles were noisier than those of *P. putida*—compounded by the high volatility of methanol (77, 78), potentially affecting the effective alcohol concentration that cells encounter in the medium.

In order to better quantify physiological responses to the alcohol challenge, shaken-flask experiments with *P. putida* EM42 were carried out in DBM medium with 20 mM glucose, with or without methanol addition at 1 M—and the concentration of substrates and potential oxidation products (e.g., formate) was measured over time (Fig. 4b). Glucose consumption increased by 20% in the presence of methanol (*q*_G_ = 6.2 ± 0.1 mmol g_CDW_^–1^ h^–1^, Fig. 4b) accounted for the extra energy obtained from methanol oxidation in addition to the standard, PQQ-dependent oxidation of glucose to gluconate in the periplasm (2). Consistent with our previous observations (Fig. 4a), the growth rate of strain EM42 was diminished by ca. 40% in methanol cultivations (Fig. 4b). We could not observe any significant reduction in methanol concentration over time, yet formate was secreted at *q*_F_ = 0.57 ± 0.03 mmol g_CDW_^–1^ h^–1^(Fig. 4b; Fig. S5)—with an increase in the acid concentration particularly noticeable by the end of the cultivation (8 h onward). This experimental evidence supports the hypothesis that formaldehyde is produced from methanol, and the aldehyde is further detoxified *via* oxidation to formate. To gain an insight into the regulatory and metabolic mechanisms underlying this methanol-sensitive phenotype, we resorted to genome-wide transcriptional fingerprinting as detailed in the next section.

### High-resolution RNA sequencing in *P*. *putida* exposed to methanol

**Fig 5:**
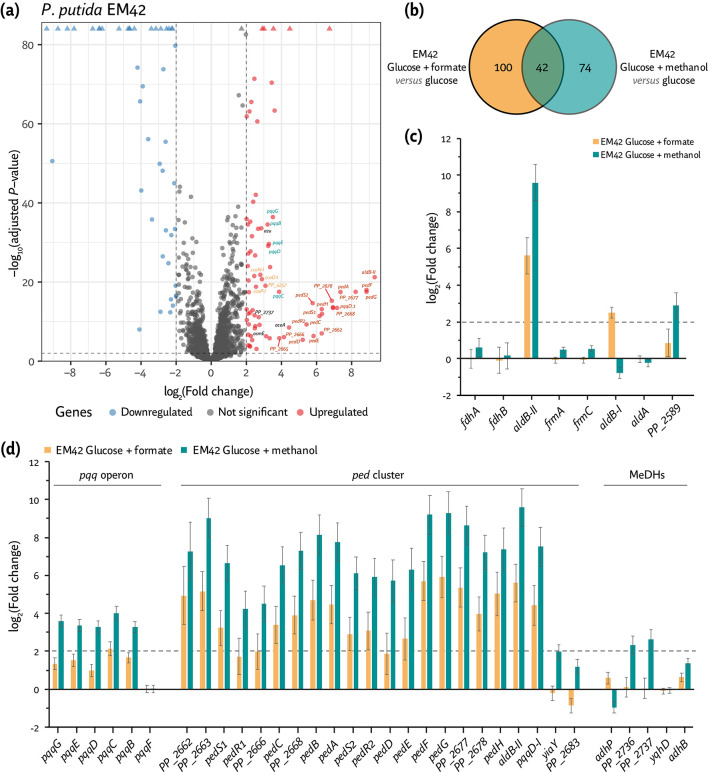
Differentially expressed genes in *P*. *putida* cultivated in the presence of methanol. (a) Volcano plot identifying differentially expressed genes (DEGs) in *P. putida* EM42 grown in de Bont minimal medium supplemented with 20 mM glucose and 1 M methanol compared to glucose cultures. Cells were harvested in the mid-exponential phase to analyze DEGs; each point in the volcano plot represents a single gene. The horizontal intersection was set at a *q*-value [FDR, false discovery rate, calculated as –log_10_(adjusted *P*-value)] ≤ 0.01 and the vertical intersections at a |log_2_(fold change)| ≥ 2. (b) DEGs in *P. putida* EM42 grown in glucose and methanol *versus* glucose compared to glucose and formate *versus* glucose. The Venn diagram shows the overlap for each pairwise comparison. (c) Log_2_(fold change) of a subset of significant encoding functions related to methanol oxidation, including characterized and putative methanol/formaldehyde dehydrogenases in the methanol and formate conditions. (d) DEGs encoded in the *pqq* operon, *ped* cluster, and putative methanol dehydrogenases (MeDHs) are highly overexpressed in the methanol condition.

In order to identify key players involved in methanol and formaldehyde oxidation in *P. putida* EM42, cells were cultivated in DBM medium containing 20 mM glucose, with or without 1 M methanol, and harvested during mid-exponential growth (5 h for cultures added with methanol) for RNA-Seq analysis (Fig. 5). Significantly DEGs between the glucose and methanol *versus* glucose-only conditions accounted for 116 ORFs [*q*-value (false discovery rate, FDR) ≤ 0.01 and |log_2_(FC)| > 2; Fig. 5a and b]. Among these DEGs, 42 genes (ca. 36%) were also present in the glucose and formate *versus* glucose comparison (Fig. 2c). Similarities in the transcriptional landscapes in formate and methanol cultivations could also be observed in a PCA of the total data. Here, the methanol and formate conditions clustered together in PC2 (with 30.1% variance) compared to the glucose-only control (Fig. S1). Indeed, the similarity between these two conditions hints at a mechanistic overlap among the detoxification strategies deployed for either C1 compound—possibly because these are intermediates within the same oxidative pathway(s). Transcriptional levels of DEGs in cells grown with methanol were compared to those in control conditions (Fig. 5a–c), testing the patterns both for their biological significance and association of the cognate functions with methanol and/or formaldehyde oxidation (Fig. 5d).

As *P. putida* can endure relatively high formaldehyde concentrations, the direct oxidative product of methanol, aldehyde detoxification systems are expected to be active upon cultivation of the cells in the presence of the C1 alcohol. Formaldehyde detoxification involves the concerted action of formaldehyde dehydrogenases and FDHs that eventually yield CO_2_ (33, 79). *P. putida* KT2440 harbors two NAD^+^-binding, glutathione-independent formaldehyde dehydrogenases (32), encoded by *fdhA* (*PP_0328*) and *fdhB* (*PP_3970*, also known as *ybdR*). A glutathione-dependent system, encoded by *frmAC* (*PP_1616-PP_1617*), is likewise present in this bacterium (61). The *frmAB* operon encodes the main formaldehyde detoxification system of *E. coli* (80 - 82), yet the role of the corresponding genes in *P. putida* remains to be studied. The FrmA and FrmC polypeptides of *P. putida* KT2440 are 72% and 53% identical to the *E. coli* K12 homologues, respectively. We initially assessed whether the detoxification systems mentioned above were differentially expressed in cells exposed to methanol compared to formate (with both conditions initially contrasted to glucose-only cultures). None of the formaldehyde dehydrogenase genes was differentially expressed in any of the C1 conditions as compared to glucose cultures (Fig. 5b and c). However, two aldehyde dehydrogenase genes, *aldB-II* [or *pedI*, *PP_2680*; log_2_(FC) = 9.6] and *PP_2589* [log_2_(FC) = 2.9], were significantly overexpressed upon methanol treatment. The nonspecific, NAD^+^-dependent dehydrogenase encoded by *aldB-I* (PP_0545), upregulated in response to ethylene glycol and butanol treatment (83), was only increased under formate conditions and slightly downregulated with methanol (Fig. 5c). These results suggest that oxidative (detoxification) activities on formaldehyde may be associated to constitutive expression of the cognate gene(s) (81)—also indicating that some of the dehydrogenase activities of *P. putida* can act on different C1 substrates.

### Multiple alcohol dehydrogenases and related functions are transcriptionally activated upon methanol treatment

We observed a considerable upregulation of the *ped* cluster (comprising *PP_2662* to *PP_2683*), the *pqq* biosynthetic operon (encoded by the *PP_0375-PP_0379* genes), as well as *PP_2736-PP_2738*, encoding a putative short-chain dehydrogenase, in cells cultivated in the presence of methanol (Fig. 5d; Table S2). The *ped* cluster encodes PedE and PedH (27), two PQQ-dependent alcohol dehydrogenases with a range (83). PedH has been identified as a lanthanide-dependent enzyme (84). YiaY, a third NAD^+^-dependent dehydrogenase recently proposed to act as a transcriptional regulator (26), is encoded within this cluster—together with the previously mentioned formaldehyde dehydrogenase gene *aldB-II* (or *pedI*). The high overexpression of *pedE* and *pedH* in the methanol condition [log_2_(FC) = 6.3 and 7.4, respectively] and the broad substrate specificity of the cognate dehydrogenases suggest a relevant role in processing the C1 alcohol. Since PedE and PedI are PQQ-dependent enzymes, it does not come as a surprise that the *pqqFABCDEG* operon was significantly upregulated in the methanol condition (Fig. 5d), with log_2_(FC) > 4. The PQQ biosynthetic pathway is required by multiple PQQ-dependent oxidases, e.g., Gcd (PP_1444), involved in the initial steps of glucose processing (85). Moreover, we analyzed different putative or characterized alcohol dehydrogenase genes, the products which could potentially act on methanol. PP_3839 (AdhP), for instance, has been previously characterized to act on short-chain alcohols (86). In our experiments, the expression of *adhP* was slightly downregulated upon methanol treatment—perhaps as a mechanism to prevent excessive formaldehyde accumulation. The putative short-chain alcohol dehydrogenases encoded by *PP_2736* and *PP_2737*, in contrast, were significantly overexpressed on methanol (Fig. 5d)—unlike *yqhD* and *adhB*, which were largely unaffected under the same conditions. Taken together, these results showcase a relatively small subset of alcohol dehydrogenase genes induced in *P. putida* by methanol treatment. Yet, other more general detoxification mechanisms were activated in response to the C1 alcohol, as detailed below.

### General detoxification mechanisms may help *P*. *putida* to cope with high methanol concentrations

We observed that formaldehyde toxicity could be partially counteracted through its oxidation to secretion (Fig. 4b), yet active aldehyde export may also contribute to detoxification. We detected overexpression of *mdtABC/ompB* (*PP_3582-PP_3585*), encoding a putative RND (resistance-nodulation-division superfamily) efflux transporter (87, 88). In contrast, the *mexEF/oprN* operon was not differentially regulated in our dataset, but a putative oxalate/formate antiporter gene (*PP_0288*) was significantly overexpressed in the methanol condition (Table S2). Also, the *cbb3*-type cytochrome oxidase encoded by the *cco-I* operon (*PP_4255-PP_4258*) was overexpressed upon methanol treatment. Such oxidases exhibit a high O_2_ affinity that allows for growth under O_2_-deprivation (89), and they are often involved in general defense mechanisms in several bacterial species (90). Paired with these highly transcribed DEGs, 47 ORFs were significantly downregulated in the methanol condition. Among them, some genes potentially involved in C1 metabolism [e.g., *sahR*, *metE*, *ahcY,* and *metF*; with log_2_(FC) < –2.9] and several uncharacterized genes, including the *dapF* diaminopimelate epimerase gene and the *aspC* aminotransferase gene [log_2_(FC) < –9.4, Table S2]. While the involvement of the cognate functions in the tolerance of *P. putida* to C1 compounds may not seem immediately evident, the transcriptional landscape discussed herein indicates a large rearrangement of gene expression patterns that involves both specific and general mechanisms. Hence, the genetic dissection of the main enzymatic activities that could process methanol and formaldehyde, as hinted in the RNA-Seq analysis, was the next step in our study.

### Redundant formaldehyde oxidation activities in *P*. *putida*

**Fig 6:**
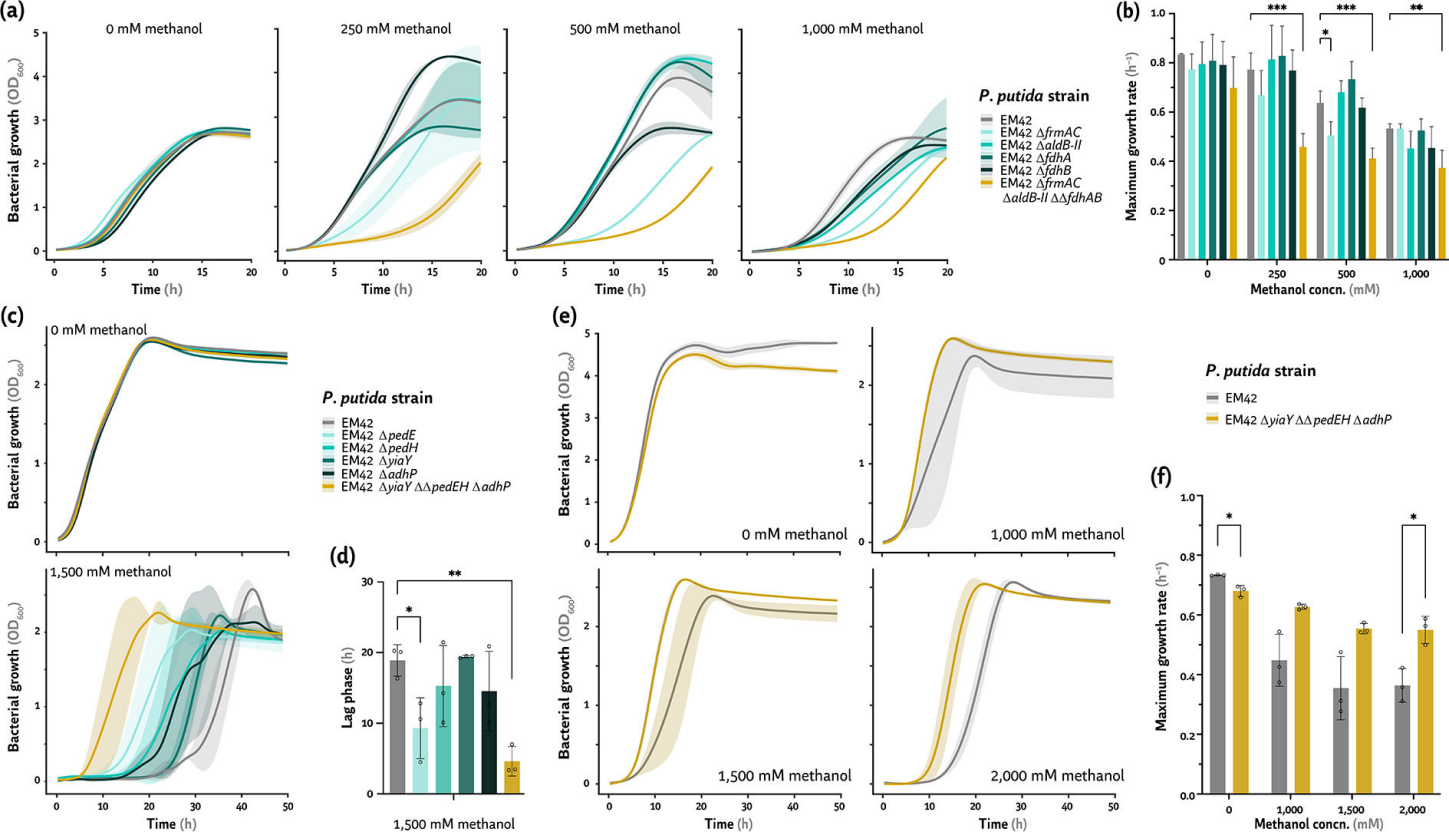
Exploring the role of formaldehyde and alcohol dehydrogenases of *P. putida* EM42 on methanol tolerance. (a and b) Growth profiles and specific growth rates of *P. putida* EM42 and the ∆*frmAC,* ∆*aldB-II,* ∆*fdhA,* ∆*fdhB,* and ∆*frmAC* ∆*aldB-II* ∆∆*fdhAB* mutants cultivated in the presence of increasing methanol concentrations (concn.). (c and d) Growth profiles and extension of the lag phase in cultures of *P. putida* EM42 and the ∆*pedE*, ∆*pedH*, ∆*yiaY*, ∆*adhP,* and ∆*yiaY* ∆∆*pedEH* ∆*adhP* mutants in the presence of absence of methanol at 1,500 mM. (e and f) Growth profiles and maximum growth rates of *P. putida* EM42 compared to its ∆*yiaY* ∆∆*pedEH* ∆*adhP* mutant derivative cultivated in the presence of increasing methanol concentrations. All bacterial cultivations were performed in de Bont minimal medium with 20 mM glucose. Average values for the bacterial growth (estimated as the optical density measured at 600 nm, OD_600_), growth rates (μ), and the extension of the lag phase (λ) ± standard deviations of three biological replicates are represented. Individual data points are shown whenever relevant. The level of significance (according to the Dunnett’s test) is indicated with asterisks as follows: * *P*-value < 0.05, ** *P*-value < 0.01, and *** *P*-value < 0.001.

Formaldehyde detoxification in *P. putida* is assumed to occur in a concerted manner, given the large number of aldehyde oxidases encoded in the genome—yet these features remained thus far understudied. The enzymes encoded by *fdhA* and its *fdhB* homologue are involved in aldehyde detoxification (32), but glutathione-independent dehydrogenases have not been identified in *P. putida*. Thus, we delved into the action of FrmAC and AldB-II, considering that the cognate genes were highly overexpressed in cells grown in the presence of methanol (Fig. 5) and the interaction of these activities with other aldehyde oxidases. Single-deletion mutants for *frmAC*, *aldB-II*, *fdhA,* and *fdhB* were constructed in the *P. putida* EM42 background, together with a quadruple mutant harboring all these knockouts (i.e., EM42 ∆*frmAC* ∆*aldB-II* ∆∆*fdhAB*, Table 1). Next, the growth profile of the mutants was compared to that of the wild-type EM42 strain in DBM medium supplemented with 20 mM glucose and increasing methanol concentrations (Fig. 6). *P. putida* ∆*frmAC* showed the starkest growth effect of all single-deletion mutants, already evident at 250 mM methanol, the lowest concentration tested (Fig. 6a). The growth rate of this strain was diminished by ca. 20% at 500 mM methanol (Fig. 6b). At higher alcohol concentrations (e.g., 1 M), all single-deletion mutants were more affected than the wild-type strain. Furthermore, the most severe growth impairment was observed in cultures of the ∆*frmAC* ∆*aldB-II* ∆∆*fdhAB* mutant, with a ca. 40% reduction in μ at 250 mM methanol when compared to *P. putida* EM42 (Fig. 6b). These results showcase synergistic effect of individual formaldehyde dehydrogenases of *P. putida* in aldehyde detoxification, with *P. putida* ∆*frmAC* as the most affected strain among all single mutants even at a low methanol concentration.

### Broad-substrate-range alcohol dehydrogenases mediate methanol oxidation in *P*. *putida*

*P. putida* KT2440 is able to oxidize a range of short-chain alcohols (91), e.g., 1-butanol (92), 1,4-butanediol (93), ethanol (26), and ethylene glycol (83, 94, 95), through the action of broad-substrate-range dehydrogenases. These activities underline the native *P. putida* preparedness to withstand toxic alcohols and aldehydes, regularly present in the natural environments where this bacterium thrives. The transcriptome of *P. putida* EM42 exposed to methanol indicated that the dehydrogenases encoded by *pedE*, *pedH,* and *adhP* (as well as the putative transcriptional regulator encoded by *yiaY*) are involved in alcohol oxidation. Hence, we constructed single-mutant strains of the above mentioned genes in strain EM42, as well as a quadruple mutant harboring the combined ∆*yiaY* ∆∆*pedEH* ∆*adhP* deletions (Table 1). Again, the growth profile of the deletion mutants was compared to that of the wild-type EM42 in DBM medium supplemented with 20 mM glucose and 1.5 M methanol (Fig. 6c), the highest alcohol concentration tolerated by all of these strains. Interestingly, all single mutants had a shorter lag phase compared to that of *P. putida* EM42, especially the ∆*pedE* strain (Fig. 6d). The quadruple ∆*yiaY* ∆∆*pedEH* ∆*adhP* knockout strain had a lag phase shorter than that of any of the single mutants (Fig. 6d), a feature that was repeated across a range of methanol concentrations (Fig. 6e). The shorter λ times were paired with increased μ values compared to *P. putida* EM42 (ca. 38% higher in the presence of 2 M methanol; Fig. 6f). Hence, the mutant strain depleted of all alcohol dehydrogenase activities tolerated a methanol challenge much better than the parental strain—confirming the hypothesis that synergistic oxidation activities of *P. putida* yield toxic formaldehyde.

## DISCUSSION

In this work, a genome-wide transcriptome analysis provided mechanistic insights into the metabolism of methanol, formaldehyde, and formate in *P. putida*. Long thought to be non-native substrates (8), these C1 molecules are processed and interconverted by a number of specific and peripheral activities in *P. putida* of formate, the most oxidized form among these soluble C1 substrates, has been assumed to occur *via* the two annotated FDHs encoded by *fdoGHI-fdhE* and *fmdEFGH* (31, 32). Our results show that, in reality, Fdo and Fmd are not the primary FDHs of *P. putida*. Upon deleting each FDH gene, either independently or jointly, similarly high *q*_F_ values were observed for all mutants in cultures containing 240 mM formate—suggesting that alternative dehydrogenases are involved in formate detoxification. Indeed, omic-guided transcriptional screening revealed that *fdoGHI-fdhE* and *fmdEFGH* were not significantly expressed under these conditions, whereas two oxidoreductase genes and *fdhD* were transcriptionally active in both the wild-type strain and the Δ*fdoGHI-fdhE* Δ*fmdEFGH* strain. Deletion of *PP_4596* alone or in combination with *fdhD* negatively affected growth in the presence of formate, providing evidence that these activities are involved in processing the C1 acid. Interestingly, PP_4596 and PP_0256 share low identity with other α-subunit components of known FDHs—yet they display high amino acid identity with Fdh4A of *M. extorquens*. Fdh4A is involved in formate oxidation (59), and overexpression of the cognate gene was observed when the cells were cultivated on methanol in the presence of 2 µM La^3+^ (96). In *M. extorquens*, *fdhB* is located right next to *fdhA*, and it was reported to be functionally coupled to Fdh4A. PP_0257, in contrast, does not share any amino acid identity with Fdh4B, and no other subunit or accessory protein was identified in relation to PP_4596—suggesting a divergent evolutionary trajectory for the *P. putida* enzymes. Mutants of *E. coli* and *Salmonella enterica*, lacking either *fdhD* or *fdhE*, display limited FDH activity (97, 98). However, almost identical ^14^CO_2_ evolution levels have been reported for *P. putida* Δ*fdhD* compared to the parental strain when fed with 10 mM ^14^C-formate or ^14^C-formaldehyde—and deleting *fdhE* hampered ^14^CO_2_ release from ^14^C-formate (33). These results indicate that the four FDHs of *P. putida* probably display different levels of substrate affinity (*K*_M_), responding to formate challenges in a concentration-dependent fashion. Interestingly, *K*_M_ values for the FDH enzyme of *Pseudomonas* sp. 101 have been shown to be influenced by the medium pH (99), adding a regulatory layer connected to the [formic acid]/[formate] ratio. Although the *in vivo* cofactor dependence of these soluble dehydrogenases remains to be explored, increased biomass yields and *pntAB* overexpression triggered by formate addition suggest that NAD^+^-dependent oxidation mechanisms prevail in *P. putida,* an NADH excess that can either be used for energy conservation or channeled into anabolism *via* transhydrogenation—a general strategy for redox balancing (70).

Formate is also the terminal C1 molecule in an oxidation sequence that starts with methanol and its direct product, formaldehyde. Hence, we also delved into the tolerance, regulation, and metabolism of *P. putida* exposed to these compounds. The genome of *P. putida* KT2440 (24) encodes 16 alcohol dehydrogenases (over half of them annotated as putative activities) as well as more than 43 aldehyde dehydrogenases (out of which three are properly characterized formaldehyde dehydrogenases). Though the *in vivo* ability of these oxidases to act on methanol or formaldehyde remains unknown, their presence underlies the native robustness of *P. putida* toward a range of toxic compounds—harnessing some of them as potential energy or carbon sources (91, 100). Our findings revealed a concerted action of both alcohol and formaldehyde dehydrogenases. Alcohol dehydrogenases mediate formaldehyde formation, decreasing methanol tolerance of *P. putida* to the C1 alcohol as compared to other organisms—e.g., *E. coli* or *Bacillus cereus. P. putida* KT2440 harbors numerous broad-substrate-range, short-chain alcohol dehydrogenases, e.g., AdhP and the PQQ-dependent enzymes encoded by *pedE* and *pedH*. PedE and PedH are highly active toward ethanol and long-chain-length alcohols, but the specific activity on methanol is relatively low at < 0.80 U mg^−1^ (27). YiaY has been assumed to encode a NAD^+^-dependent methanol dehydrogenase, and overexpressing the cognate gene resulted in increased methanol consumption (28). However, YiaY was recently hypothesized to function as the regulatory partner of a two-component sensor system, given its sequence similarity to the well-characterized *ercA* gene of *P. aeruginosa* PAO1 (26). Hence, YiaY would sense methanol through its oxidation, generating a signal that activates the putative histidine kinase PP_2683. Indeed, deleting *yiaY* in *P. putida* lowered *pedH* and *aldB-II* expression upon an ethanol challenge (26). In line with these interpretations, we have observed reduced methanol toxicity for *P. putida* ∆*yiaY*, likely due to low expression of the abovementioned genes and leading to limited formaldehyde formation. Furthermore, the combined deletion of ∆*yiaY* ∆∆*pedEH* and ∆*adhP* resulted in the highest methanol resistance and growth rates compared to the parental EM42 strain across a range of alcohol concentrations.

Formaldehyde detoxification systems may be constitutive or inducible (101). In *P. putida* KT2440, *fdhB* (*PP_3970*) has been proposed to be constitutively expressed (61, 79), yet the *frmAB* operon of *E. coli* is induced by the FrmR regulator (80, 102). We observed a similarly constitutive expression pattern for *fdhAB* in our study, whereas *aldB-II* represented the highest upregulated gene of the whole dataset. Interestingly, the expression of the *frmAC* operon was not induced significantly as in *E. coli*. The regulator to *frmAC* in *P. putida* (PP_1615) has no significant homology to FrmR of *E. coli* K12—but shares >80% amino acid homology with GfnR (PA_3630) of *P. aeruginosa*. The GfnR protein has been characterized to be activated upon sarcosine (*N*-methylglycine) supplementation, thereby upregulating the glutathione-dependent detoxification system encoded in the *adhC*/*PA_3628* operon (102, 103). Sarcosine is converted into glycine and formaldehyde *via* oxidative demethylation catalyzed by sarcosine oxidase (104). FrmA and FrmC have an 86% and 77% amino acid similarity to their *P. aeruginosa* orthologues, respectively. As we observed a severe growth defect for the ∆*frmAC* mutant of *P. putida* EM42 even at the lowest methanol concentration, we hypothesize that the FrmAC detoxification system, regulated by PP_1615, may be the most active to deal with low formaldehyde levels. In contrast, the concerted action of multiple enzymes seems to detoxify higher aldehyde concentrations. We also conclude that AldB-II (PedI) may be involved in formaldehyde oxidation at high concentrations, given the growth impairment of the corresponding deletion mutant and the broad substrate specificity of alcohol and aldehyde dehydrogenases encoded in the *ped* gene cluster. Finally, we note that a C1 transfer pathway, linked to tetrahydrofolate (THF) metabolism and widespread in Nature (105), exists in *P. putida* KT2440. This route involves the bifunctional FolD enzyme, possessing both methylene-THF dehydrogenase and methenyl-THF cyclohydrolase activities, and the non-reversible formyl-THF hydrolase (PurU) enzyme. Although we cannot rule out that FolD/PurU participate in formaldehyde processing by *P. putida*, the cognate genes could not be identified in the transcriptome datasets.

**Fig 7:**
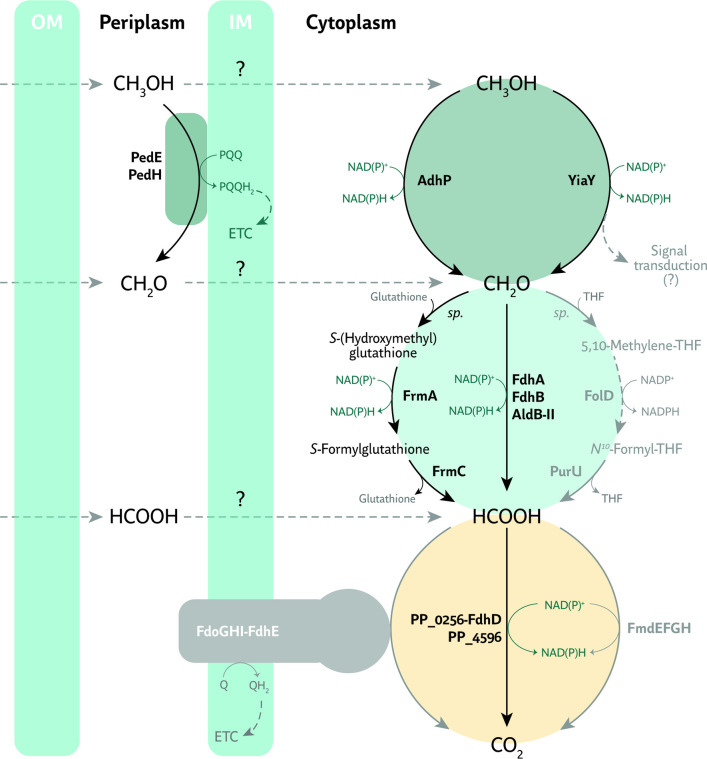
Updated model of the native routes for one-carbon metabolism in *P. putida*. Methanol (CH_3_OH), formaldehyde (CH_2_O), and formate (HCOOH) can enter the bacterial periplasm either *via* porines or by diffusing through the outer membrane (OM). Methanol, the most reduced form of these chemical species, can either (i) be oxidized to formaldehyde by the pyrroloquinoline quinone (PQQ)-dependent alcohol dehydrogenases PedE and/or PedH in the periplasm or (ii) penetrate the inner membrane (IM), to be oxidized in the cytoplasm by other broad-substrate-range dehydrogenases, e.g., the NAD(P)^+^-dependent AdhP or YiaY proteins. The latter may also induce a signal transduction cascade to transcriptionally activate the *ped* cluster. Formaldehyde, in turn, can be oxidized to formate through different pathways, e.g., the glutathione-dependent (FrmAC) and the thiol-independent (FdhA, FdhB, and AldB-II) mechanisms or the tetrahydrofolate methylation loop. Finally, formate may be fully oxidized to CO_2_ *via* the NAD(P)^+^-dependent PP_0256-0257 and PP_4596 activities reported in this work or, to a lesser extent, *via* FdoGHI-FdhE and FmdEFGH. The transport mechanisms through bacterial membranes for one-carbon molecules are yet unknown (indicated with a “?” symbol). Electrons harvested by quinones (Q) or PQQ may directly be channeled into the electron transport chain (ETC) for energy conservation. *sp*., spontaneous.

Taken together, our results enabled us to propose an updated model for the metabolism of C1 compounds in *P. putida* (Fig. 7). Besides expanding the current understanding of how microbes process methanol, formaldehyde, and formate, our study showcases the possibility of valorizing C1 feedstocks by engineering the versatile routes native to *P. putida*. While further research will help elucidate the mechanism of action for the novel activities reported herein, our work serve as a cornerstone toward establishing synthetic methylotrophy in *P. putida*. Meanwhile, we envisage this soil bacterium to be a favored microbial host for metabolic engineering of C1 assimilation given its natural ability to process methanol and its direct oxidation products, formaldehyde and formate.

## Data Availability

All the raw RNA sequencing data generated in this study are publicly available at the NCBI database with accession number PRJNA955793.

## SUPPLEMENTAL MATERIAL

Figure S1 msystems.00004-23-s0001.pdf Statistical analysis of the RNA-Seq data in this study.

Figure S2 msystems.00004-23-s0002.pdf Amino acid identity between PP_0256, PP_4596, and Fdh4A.

Figure S3 msystems.00004-23-s0003.pdf Amino acid identity between PP_0256, PP_4596, Fdh4A, and YdeP.

Figure S4 msystems.00004-23-s0004.pdf Amino acid identity between PP_0256 and PP_4596.

Figure S5 msystems.00004-23-s0005.pdf Exometabolome fingerprint of *P. putida* EM42 cultivated in the presence of methanol.

TABLE S1 msystems.00004-23-s0006.pdf Changes in gene expression of *P. putida* EM42 grown in the presence of different C1 substrates.

TABLE S2 msystems.00004-23-s0007.pdf Gene ontology analysis for *P. putida* EM42 genes differentially expressed in the methanol and formate conditions compared to the glucose-only control.

TABLE S3 msystems.00004-23-s0008.pdf Oligonucleotides used in this study.

ASM does not own the copyrights to Supplemental Material that may be linked to, or accessed through, an article. The authors have granted ASM a non-exclusive, world-wide license to publish the Supplemental Material files. Please contact the corresponding author directly for reuse.
