# Supplementary material for: Core and auxiliary functions of one-carbon metabolism in Pseudomonas putida exposed by a systems-level analysis of transcriptional and physiological responses
Source: mSystems. 2023 Jun 5;8(3):e00004-23. doi: 10.1128/msystems.00004-23 (PMC10308882; doi:10.1128/msystems.00004-23)
Supplement: Figure S1 — Statistical analysis of the RNA-Seq data in this study. [file msystems.00004-23-s0001.pdf]

Fig. S1. Statistical analysis of the RNA-Seq data in this study.

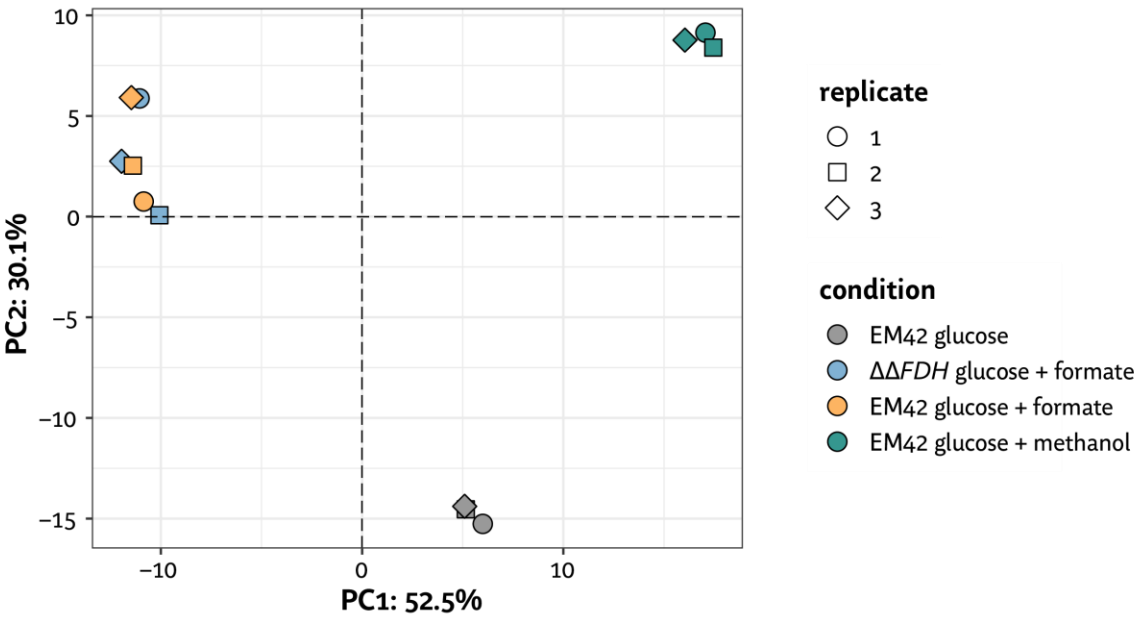

PCA (principal component analysis) was performed for quality control purposes. The percentage of the variance explained by principal component (PC) 1 is 52.5%, while the percentage of variance explained by PC 2 is 30.1%. The percentage of the variance explained by the two first PCs amounts to 82.6%.
