# Supplementary material for: Core and auxiliary functions of one-carbon metabolism in Pseudomonas putida exposed by a systems-level analysis of transcriptional and physiological responses
Source: mSystems. 2023 Jun 5;8(3):e00004-23. doi: 10.1128/msystems.00004-23 (PMC10308882; doi:10.1128/msystems.00004-23)
Supplement: Figure S2 — Amino acid identity between PP_0256, PP_4596, and Fdh4A. [file msystems.00004-23-s0002.pdf]

Fig. S2. Amino acid identity between PP_0256, PP_4596 and Fdh4A.

```
Fdh4A           -----------MDRSQGLPKRSSAAGGWGALKSCGKFLLGSRAPISGARALLSANQPDGF    49
PP_0256         MTSYQQLPDNTPASPPRYKPYHGPAGGWGALRSVAKAWVGSDNALKNIRALLKTNQNGGF    60
PP_4596         -----------MSQDEHIRDYKGAAAGWGALKSVTKSWLGSDNAFKNLRAMLKTNQNGGF    49
                                      . *.*****:*  *  :**   :.. **:*.:** .**

Fdh4A           DCPGCAWGDPAHGSSFEFCENGVKAVSWEATDKRATPRFFAKHPVSELRGWTDYALESEG    109
PP_0256         DCPGCAWGDSPESGMVKFCENGAKAVNWEATKRRVDAAFFARYSVTSLLQQSDYWLEYQG    120
PP_4596         DCPGCAWGESPESDMVKFCENGAKAVNWEATGRSVDPAFFAKYSVSALKEQTDYWLEYQG    109
                ********:  ... .:*****.***.**** : .   ***:: *: *   :** ** :*

Fdh4A           RLTHPMRYDAETDTYRAVEWDEAFAEIGATLRSLDHPDRVEFYTSGRASNEAAYLYQLFA    169
PP_0256         RLTEPMVYDAPSDRYLPISWDAAFALIARELNKLANPDQAEFYTSGRASNEAAYLYQLFV    180
PP_4596         RLTHPMRYDAATDHYVETTWQEAFELVARHLRALQSPDEAEFYTSGRASNEAAFLYQLFV    169
                ***.** *** :* *    *: **  :.  *. *  **..*************:*****.

Fdh4A           RAYGTNNFPDCSNMCHEASGIALVQAIGIGKGTVLLEDFEKADAIFVVGQNPGTNHPRML    229
PP_0256         RAYGTNNFPDCSNMCHEASGVALGQSVGVGKGTVTFDDFEHADAIFVWGQNPGTNHPRML    240
PP_4596         RAYGTNNFPDCSNMCHEASGAGMSETLGVGKGTVVFHDLELADAIFVIGQNPGTNHPRML    229
                ******************** .: :::*:***** :.*:* ****** ************

Fdh4A           GDLRRAAERGARVVVLNPVRERGLERFADPQNSVEMLRGASRPIASHYFQPKPGGDMAAF    289
PP_0256         DPLRDAVKRGAQVVCINPLKERGLERFQHPQNPLEMLTNSDRPTNTAFFRPALGGDMAML    300
PP_4596         EPLREAVKRGAQVVCFNPLKERGLERFQHPQHPFEMLSNGSEPTSSAYFRPALGGDMAAM    289
                  ** *.:***:** :**::******* .**: .*** ....*  : :*:*  ***** :

Fdh4A           RGIAKVVFARDAAAIEAGKPSLLDHAFIAAHTSAFADYRAAVETTAWDAILDQSGLTREE    349
PP_0256         RGMAKFVLQWEREAQANGEPAVFDHAFIAEHGHGVDEYLAVVDATPWSHIQAQSGLTLAD    360
PP_4596         RGIAKYLLQWEREAQAKGEPAVFDHAFIAEHTSGVDDYLAAVDATSWEHIVKQSGLTLAE    349
                **:** ::  :  *   *:*:::****** *  .. :* *.*::* *. *  *****  :

Fdh4A           IETAADVYLGADKVIATWAMGVTQHRHSVATIREIANLLFLRGHIGRPGAGLCPVRGHSN    409
PP_0256         IELAARMYCQGKRVIMCWAMGITQHRHSVPTIQEIVNLQMLRGNIGVPGAGLCPVRGHSN    420
PP_4596         IELAARMYRKAERVIMCWAMGVTQHRHSVPTVQEIVNLQLLRGNVGKPGAGLSPVRGHSN    409
                ** ** :*  ..:**  ****:******* *::**.** :***::* *****.*******

Fdh4A           VQGDRTVGINEKPPLALLEALDREFGLNIPRKHGHNVLGAIGAMLDGSAKAFIGLGGNFV    469
PP_0256         VQGDRTMGINERPPEALLDAIEKRFGFPVPRRNGHNTVEAIHAMLDGRAKVFIGLGGNFA    480
PP_4596         VQGDRTMGIDEKPSAALLDAIEQRFQFSVPRTHGHNAVLAIKAMEEGRAKVFIGLGGNFA    469
                ******:**:*:*  ***:*:::.* : :** :***.: ** ** :* **.********.

Fdh4A           RATPDTRLVEKALAGCELTVHIATKLNHSHLVPGRVSYLLPCLGRTEIDRNSRAKVQIVT    529
PP_0256         QATPDTERTAQALRNCELTVHISTKLNRSHLVHGKQALILPCLGRTDIDLQADG-PQAVT    539
PP_4596         QATPDTARTHAALQNCALTVQISTKLNRSHLITGRDALILPCLGRTEIDLQAEG-PQGVT    528
                :*****  .  ** .* ***:*:****:***: *: : :*******:** :: .  * **

Fdh4A           VEDSMSMVHGSGGINKPASPHLRSEIGIIAGMAAATVGSERIDWAALADDYDLIRDRIER    589
PP_0256         VEDSFSMVHASNGQLKPLSTQMRSEPAVIAGIAAATLGKQPVDWHWLVADYDRIRDLIGD    599
PP_4596         VEDTFSMVHISNGQLRPRSPHMRSEPWIIAGMAKATLGNQPIDWEYAVADYNRIRDMIAD    588
                ***::**** *.*  :* * ::***  :***:* **:*.: :**   . **: *** *

Fdh4A           TIPGFSGFNTRVRRPRGFMLRNLAAERVFETATGRAGFSSGPLPVATEHQRASLRG--DT    647
PP_0256         TIPGFSGFNQRLRNPGGFYLGNSAASREWATSTGRANFKANLLPDTLLDERVRASGQLPD    659
PP_4596         VIPGFTGFNERLNSPGGFHLGNNAADRNFRTATGKARFMPHALPEELVNAKVLARGDKPD    648
                .****:*** *:. * ** * * **.* : *:**:* *    **    . :.   *

Fdh4A           FVLQTFRSHDQYNTTIYGLDDRYRGVYGERRVVFANPDDLAELKARAGERVDLVCVHAED    707
PP_0256         LIMQSMRSHDQYNTTIYGLDDRYRGVRGQREVLFANEADIIRLGFQPGQKVDIVSLWG-D    718
PP_4596         LILQTLRSHDQYNTTLYGLDDRYRGVFGLREVVFVNEADIRRLGFEPGEQVDLVSLWE-D    707
                :::*::*********:********** * *.*:*.*  *: .*  . *::**:*.:   *
```

```
Fdh4A           GVERVAEDFRLVPFDMPRGALAGYYPELNVLVPLSAFGEFSDTPTSKSVLVQVRARAAND    767
PP_0256         EHVRRVQGFTLLAFDIPAGQAAAYYPEVNPLVPLESIGVGSHTPTSKFIAIKLERAREDG    778
PP_4596         GVERRVSGFRLVAYDVPEGQAAAYYPETNPLVPLESYGEGTYTPTSKFVAIKVEKAKAGN    767
                   * ...* *: :*:* *  *.**** * ****.: *  : ***** : :::.    ..

Fdh4A           LGKAA----    772
PP_0256         RIL------    781
PP_4596         RIAAVLASD    776
```

Protein sequence alignment of PP_0256, PP_4596 and Fdh4A with Clustal Omega. An asterisk symbol (*) indicates positions which have a single, fully conserved residue. A colon (:) indicates conservation between groups of strongly similar properties. A period (.) indicates conservation between groups of weakly similar properties.
