# Supplementary material for: Core and auxiliary functions of one-carbon metabolism in Pseudomonas putida exposed by a systems-level analysis of transcriptional and physiological responses
Source: mSystems. 2023 Jun 5;8(3):e00004-23. doi: 10.1128/msystems.00004-23 (PMC10308882; doi:10.1128/msystems.00004-23)
Supplement: Figure S3 — Amino acid identity between PP_0256, PP_4596, Fdh4A, and YdeP. [file msystems.00004-23-s0003.pdf]

Fig. S3. Amino acid identity between PP_0256, PP_4596, Fdh4A and YdeP.

```
YdeP         -------------MKKKIESYQGAAGGWGAVKSVANAVRKQMDIRQDVIAMFDMNKPEGF  47
Fdh4A        -----------MDRSQGLPKRSSAAGGWGALKSCGKFLLGSRAPISGARALLSANQPDGF  49
PP_0256      MTSYQQLPDNTPASPPRYKPYHGPAGGWGALRSVAKAWVGSDNALKNIRALLKTNQNGGF  60
PP_4596      -----------MSQDEHIRDYKGAAAGWGALKSVTKSWLGSDNAFKNLRAMLKTNQNGGF  49
                                   . *.****::*  :    .    ..  *::. *:  **

YdeP         DCPGCAWPDPKHSASFDICENGAKAIAWEVTDKQVNASFFAENTVQSLLTWGDHELEAAG  107
Fdh4A        DCPGCAWGDPAHGSSFEFCENGVKAVSWEATDKRATPRFFAKHPVSELRGWTDYALESEG  109
PP_0256      DCPGCAWGDSPESGMVKFCENGAKAVNWEATKRRVDAAFFARYSVTSLLQQSDYWLEYQG  120
PP_4596      DCPGCAWGESPESDMVKFCENGAKAVNWEATGRSVDPAFFAKYSVSALKEQTDYWLEYQG  109
            ******* :  ..  ..:****.**: **.* : .   ***.  *  *    *: **  *

YdeP        RLTQPLKYDAVSDCYKPLSWQQAFDEIGARLQSYSDPNQVEFYTSGRTSNEAAFLYQLFA  167
Fdh4A       RLTHPMRYDAETDTYRAVEWDEAFAEIGATLRSLDHPDRVEFYTSGRASNEAAYLYQLFA  169
PP_0256     RLTEPMVYDAPSDRYLPISWDAAFALIARELNKLANPDQAEFYTSGRASNEAAYLYQLFV  180
PP_4596     RLTHPMRYDAATDHYVETTWQEAFELVARHLRALQSPDEAEFYTSGRASNEAAFLYQLFV  169
            ***.*: *** :* *    *: **  :.  *.    *:..*******:*****:*****.

YdeP        REYGSNNFPDCSNMCHEPTSVGLAASIGVGKGTVLLEDFEKCDLVICIGHNPGTNHPRML  227
Fdh4A       RAYGTNNFPDCSNMCHEASGIALVQAIGIGKGTVLLEDFEKADAIFVVGQNPGTNHPRML  229
PP_0256     RAYGTNNFPDCSNMCHEASGVALGQSVGVGKGTVTFDDFEHADAIFVWGQNPGTNHPRML  240
PP_4596     RAYGTNNFPDCSNMCHEASGAGMSETLGVGKGTVVFHDLELADAIFVIGQNPGTNHPRML  229
            * **:************ :. .:  ::*:***** :.*:* .* ::  *:**********

YdeP        TSLRALVKRGAKMIAINPLQERGLERFTAPQNPFEMLTNSETQLASAYYNVRIGGDMALL  287
Fdh4A       GDLRRAAERGARVVVLNPVRERGLERFADPQNSVEMLRGASRPIASHYFQPKPGGDMAAF  289
PP_0256     DPLRDAVKRGAQVVCINPLKERGLERFQHPQNPLEMLTNSDRPTNTAFFRPALGGDMAML  300
PP_4596     EPLREAVKRGAQVVCFNPLKERGLERFQHPQHPFEMLSNGSEPTSSAYFRPALGGDMAAM  289
              **  .:***::: :**::*******  **: .*** ...    : ::.   ***** :

YdeP        KGMMRLLIERDDAASAAGRPSLLDDEFIQTHTVGFDELRRDVLNSEWKDIERISGLSQTQ  347
Fdh4A       RGIAKVVFARDAAAIEAGKPSLLDHAFIAAHTSAFADYRAAVETTAWDAILDQSGLTREE  349
PP_0256     RGMAKFVLQWEREAQANGEPAVFDHAFIAEHGHGVDEYLAVVDATPWSHIQAQSGLTLAD  360
PP_4596     RGIAKYLLQWEREAQAKGEPAVFDHAFIAEHTSGVDDYLAAVDATSWEHIVKQSGLTLAE  349
            :*: : ::  :  *   *.*:::*. **  *  .. :    *  : *. *   ***:  :

YdeP        IAELADAYAAAERTIICYGMGITQHEHGTQNVQQLVNLLLMKGNIGKPGAGICPLRGHSN  407
Fdh4A       IETAADVYLGADKVIATWAMGVTQHRHSVATIREIANLLFLRGHIGRPGAGLCPVRGHSN  409
PP_0256     IELAARMYCQGKRVIMCWAMGITQHRHSVPTIQEIVNLQMLRGNIGVPGAGLCPVRGHSN  420
PP_4596     IELAARMYRKAERVIMCWAMGVTQHRHSVPTVQEIVNLQLLRGNVGKPGAGLSPVRGHSN  409
            *   *  *  ..:.*  :.**:***.*.. .::::.** :::*::* ****:.*:*****

YdeP        VQGDRTVGITEKPSAEFLARLGERYGFTPPHAPGHAAIASMQAICTGQARALICMGGNFA  467
Fdh4A       VQGDRTVGINEKPPLALLEALDREFGLNIPRKHGHNVLGAIGAMLDGSAKAFIGLGGNFV  469
PP_0256     VQGDRTMGINERPPEALLDAIEKRFGFPVPRRNGHNTVEAIHAMLDGRAKVFIGLGGNFA  480
PP_4596     VQGDRTMGIDEKPSAALLDAIEQRFQFSVPRTHGHNAVLAIKAMEEGRAKVFIGLGGNFA  469
            ******:** *:*   :*  : ..: :  *:  ** .: :: *:  * *:.:* :****.
YdeP        LAMPDREASAVPLTQLDLAVHVATKLNRSHLLTARHSYILPVLGRSEIDMQKNG-AQAVT  526
Fdh4A       RATPDTRLVEKALAGCELTVHIATKLNHSHLVPGRVSYLLPCLGRTEIDRNSRAKVQIVT  529
PP_0256     QATPDTERTAQALRNCELTVHISTKLNRSHLVHGKQALILPCLGRTDIDLQADG-PQAVT  539
PP_4596     QATPDTARTHAALQNCALTVQISTKLNRSHLITGRDALILPCLGRTEIDLQAEG-PQGVT  528
             * **       *    *:*:::****:***: .: : :** ***::** :  .  * **

YdeP        VEDSMSMIHASRGVLKPAGVMLKSECAVVAGIAQAALPQSVVAWEYLVEDYDRIRNDIEA  586
Fdh4A       VEDSMSMVHGSGGINKPASPHLRSEIGIIAGMAAATVGSERIDWAALADDYDLIRDRIER  589
PP_0256     VEDSFSMVHASNGQLKPLSTQMRSEPAVIAGIAAATLGKQPVDWHWLVADYDRIRDLIGD  599
PP_4596     VEDTFSMVHISNGQLRPRSPHMRSEPWIIAGMAKATLGNQPIDWEYAVADYNRIRDMIAD  588
            ***::**:* * *  :* .  ::**  ::**:* *:: .. : *   . **: **: *

YdeP        VLPEFADYNQRIRHPGGFHLINAAAERRWMTPSGKANFITSKGL---LEDP--SSAFNSK  641
Fdh4A       TIPGFSGFNTRVRRPRGFMLRNLAAERVFETATGRAGFSSGPLPVATEHQRASLRG--DT  647
```

```
PP_0256       TIPGFSGFNQRLRNPGGFYLGNSAASREWATSTGRANFKANLLPDTLLDERVRASGQLPD   659
PP_4596       VIPGFTGFNERLNSPGGFHLGNNAADRNFRTATGKARFMPHALPEELVNAKVLARGDKPD   648
              .:* *:.:* *:. * ** * * **.* : * :*:* *          .      .

YdeP          LVMATVRSHDQYNTTIYGMDDRYRGVFGQRDVVFMSAKQAKICRVKNGERVNLIALTPDG   701
Fdh4A         FVLQTFRSHDQYNTTIYGLDDRYRGVYGERRVVFANPDDLAELKARAGERVDLVCVHAED   707
PP_0256       LIMQSMRSHDQYNTTIYGLDDRYRGVRGQREVLFANEADIIRLGFQPGQKVDIVSLWG-D   718
PP_4596       LILQTLRSHDQYNTTLYGLDDRYRGVFGLREVVFVNEADIRRLGFEPGEQVDLVSLWE-D   707
              ::: :.*********:**:******* * * *:* .  :      . *::*:::.:   .

YdeP          KRSSRRMDRLKVVIYPMADRSLVTYFPESNHMLTLDNHDPLSGIPGYKSIPVELEPSN--   759
Fdh4A         -GVERVAEDFRLVPFDMPRGALAGYYPELNVLVPLSAFGEFSDTPTSKSVLVQVRARAAN   766
PP_0256       -EHVRRVQGFTLLAFDIPAGQAAAYYPEVNPLVPLESIGVGSHTPTSKFIAIKLERARED   777
PP_4596       -GVERRVSGFRLVAYDVPEGQAAAYYPETNPLVPLESYGEGTYTPTSKFVAIKVEKAKAG   766
                   *  . : :: : :     . *:** * :: *.  .  :  *  * : :::.

YdeP          ----------     759
Fdh4A         DLGKAA----     772
PP_0256       GRIL------     781
PP_4596       NRIAAVLASD     776
```

Protein sequence alignment of PP_0256, PP_4596 and Fdh4A with Clustal Omega. An asterisk symbol (*) indicates positions which have a single, fully conserved residue. A colon (:) indicates conservation between groups of strongly similar properties. A period (.) indicates conservation between groups of weakly similar properties.
