# Supplementary material for: Core and auxiliary functions of one-carbon metabolism in Pseudomonas putida exposed by a systems-level analysis of transcriptional and physiological responses
Source: mSystems. 2023 Jun 5;8(3):e00004-23. doi: 10.1128/msystems.00004-23 (PMC10308882; doi:10.1128/msystems.00004-23)
Supplement: Figure S4 — Amino acid identity between PP_0256 and PP_4596. [file msystems.00004-23-s0004.pdf]

Fig. S4. Amino acid identity between PP_0256 and PP_4596.

```
PP_0256      MTSYQQLPDNTPASPPRYKPYHGPAGGWGALRSVAKAWVGSDNALKNIRALLKTNQNGGF   60
PP_4596      -----------MSQDEHIRDYKGAAAGWGALKSVTKSWLGSDNAFKNLRAMLKTNQNGGF   49
                         :.  : : *:* *.*****:**:*:*:*****:**:**:*********

PP_0256      DCPGCAWGDSPESGMVKFCENGAKAVNWEATKRRVDAAFFARYSVTSLLQQSDYWLEYQG   120
PP_4596      DCPGCAWGESPESDMVKFCENGAKAVNWEATGRSVDPAFFAKYSVSALKEQTDYWLEYQG   109
             ********:****.***************** * ** ****:***::* :*:********

PP_0256      RLTEPMVYDAPSDRYLPISWDAAFALIARELNKLANPDQAEFYTSGRASNEAAYLYQLFV   180
PP_4596      RLTHPMRYDAATDHYVETTWQEAFELVARHLRALQSPDEAEFYTSGRASNEAAFLYQLFV   169
             ***.** *** :*:*:  :*: ** *:**.*. * .**:**************:******

PP_0256      RAYGTNNFPDCSNMCHEASGVALGQSVGVGKGTVTFDDFEHADAIFVWGQNPGTNHPRML   240
PP_4596      RAYGTNNFPDCSNMCHEASGAGMSETLGVGKGTVVFHDLELADAIFVIGQNPGTNHPRML   229
             ********************..:.:::*******.*.*:* ****** ************

PP_0256      DPLRDAVKRGAQVVCINPLKERGLERFQHPQNPLEMLTNSDRPTNTAFFRPALGGDMAML   300
PP_4596      EPLREAVKRGAQVVCFNPLKERGLERFQHPQHPFEMLSNGSEPTSSAYFRPALGGDMAAM   289
             :***:**********:***************:*:***:*...**.:*:********** :

PP_0256      RGMAKFVLQWEREAQANGEPAVFDHAFIAEHGHGVDEYLAVVDATPWSHIQAQSGLTLAD   360
PP_4596      RGIAKYLLQWEREAQAKGEPAVFDHAFIAEHTSGVDDYLAAVDATSWEHIVKQSGLTLAE   349
             **:**::*********:**************  ***:***.**** *.**  *******:

PP_0256      IELAARMYCQGKRVIMCWAMGITQHRHSVPTIQEIVNLQMLRGNIGVPGAGLCPVRGHSN   420
PP_4596      IELAARMYRKAERVIMCWAMGVTQHRHSVPTVQEIVNLQLLRGNVGKPGAGLSPVRGHSN   409
             ******** :.:*********:*********:*******:****:* *****.*******

PP_0256      VQGDRTMGINERPPEALLDAIEKRFGFPVPRRNGHNTVEAIHAMLDGRAKVFIGLGGNFA   480
PP_4596      VQGDRTMGIDEKPSAALLDAIEQRFQFSVPRTHGHNAVLAIKAMEEGRAKVFIGLGGNFA   469
             *********:*:*  *******:** * *** :***:* **:** :**************

PP_0256      QATPDTERTAQALRNCELTVHISTKLNRSHLVHGKQALILPCLGRTDIDLQADGPQAVTV   540
PP_4596      QATPDTARTHAALQNCALTVQISTKLNRSHLITGRDALILPCLGRTEIDLQAEGPQGVTV   529
             ****** **  **:** ***:**********: *::**********:*****:***.***

PP_0256      EDSFSMVHASNGQLKPLSTQMRSEPAVIAGIAAATLGKQPVDWHWLVADYDRIRDLIGDT   600
PP_4596      EDTFSMVHISNGQLRPRSPHMRSEPWIIAGMAKATLGNQPIDWEYAVADYNRIRDMIADV   589
             **:***** *****:* * :***** :***:* ****:**:**.: ****:****:*.*.

PP_0256      IPGFSGFNQRLRNPGGFYLGNSAASREWATSTGRANFKANLLPDTLLDERVRASGQLPDL   660
PP_4596      IPGFTGFNERLNSPGGFHLGNNAADRNFRTATGKARFMPHALPEELVNAKVLARGDKPDL   649
             ****:***:**..****:***.**.*:: *:**:*.*  : **: *:: :* * *: ***

PP_0256      IMQSMRSHDQYNTTIYGLDDRYRGVRGQREVLFANEADIIRLGFQPGQKVDIVSLWGDEH   720
PP_4596      ILQTLRSHDQYNTTLYGLDDRYRGVFGLREVVFVNEADIRRLGFEPGEQVDLVSLWEDGV   709
             *:*::*********:********** * ***:*.***** ****:**::**:**** *

PP_0256      VRRVQGFTLLAFDIPAGQAAAYYPEVNPLVPLESIGVGSHTPTSKFIAIKLERAREDGRI   780
PP_4596      ERRVSGFRLVAYDVPEGQAAAYYPETNPLVPLESYGEGTYTPTSKFVAIKVEKAKAGNRI   769
              ***.** *:*:*:* *********.******** * *::******:***:*:*: ..**

PP_0256       L------       781
PP_4596       AAVLASD       776
```

Protein sequence alignment of PP_0256, PP_4596 and Fdh4A with Clustal Omega. An asterisk symbol (*) indicates positions which have a single, fully conserved residue. A colon (:) indicates conservation between groups of strongly similar properties. A period (.) indicates conservation between groups of weakly similar properties.
