# Supplementary material for: Core and auxiliary functions of one-carbon metabolism in Pseudomonas putida exposed by a systems-level analysis of transcriptional and physiological responses
Source: mSystems. 2023 Jun 5;8(3):e00004-23. doi: 10.1128/msystems.00004-23 (PMC10308882; doi:10.1128/msystems.00004-23)
Supplement: Figure S5 — Exometabolome fingerprint of P. putida EM42 cultivated in the presence of methanol. [file msystems.00004-23-s0005.pdf]

Fig. S5. Exometabolome fingerprint of *P. putida* EM42 cultivated in the presence of methanol.

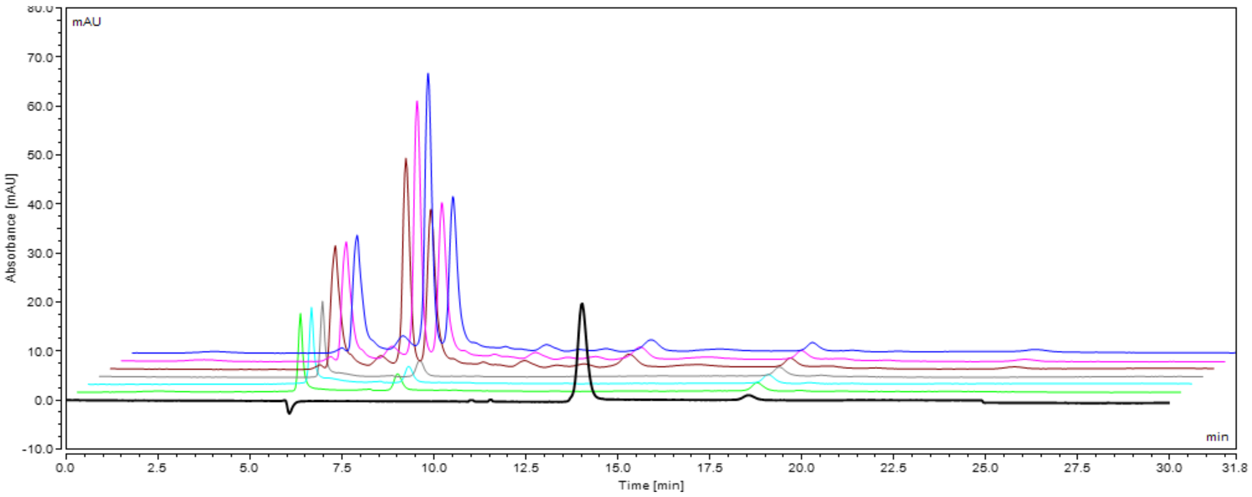

HPLC chromatograms of supernatants from strain EM42 cultivated with glucose and methanol after 10.5 h are shown with different colors; an authentic formate standard is indicated in black.
