# Supplementary material for: Core and auxiliary functions of one-carbon metabolism in Pseudomonas putida exposed by a systems-level analysis of transcriptional and physiological responses
Source: mSystems. 2023 Jun 5;8(3):e00004-23. doi: 10.1128/msystems.00004-23 (PMC10308882; doi:10.1128/msystems.00004-23)
Supplement: TABLE S1 — Changes in gene expression of P. putida EM42 grown in the presence of different C1 substrates. [file msystems.00004-23-s0006.pdf]

Table S1. Changes in gene expression of *P. putida* EM42 grown in the presence of different C1 substrates.

Gene ID	Locus	Gene	ΔΔFDH glucose + formate *versus* EM42 glucose		EM42 glucose + formate *versus* EM42 glucose		EM42 glucose + methanol *versus* EM42 glucose	
			Log_2(FC)	SE	Log_2(FC)	SE	Log_2(FC)	SE
**PP_RS24220**	PP_4649	*PP_4649*	6.3	0.3	6.5	0.3	0.6	0.4
**PP_RS23935**	PP_4596	*PP_4596*	5.8	0.3	5.9	0.3	–0.2	0.4
**PP_RS10660**	PP_2052	*PP_2052*	5.8	0.2	5.7	0.2	–0.5	0.2
**PP_RS24225**	PP_4650	*cydB*	5.6	0.2	5.9	0.2	0.2	0.3
**PP_RS13935**	PP_2676	*PP_2676*	5.4	1.1	5.9	1.1	9.3	1.1
**PP_RS13930**	PP_2675	*pedF*	5.4	1.0	5.7	1.0	9.2	1.0
**PP_RS24230**	PP_4651	*PP_4651*	5.4	0.2	5.7	0.2	–0.1	0.3
**PP_RS13955**	PP_2680	*PP_2680*	5.3	1.0	5.6	1.0	9.6	1.0
**PP_RS13940**	PP_2677	*PP_2677*	5.0	1.0	5.4	1.0	8.6	1.0
**PP_RS13950**	PP_2679	*pedH*	4.8	1.1	5.0	1.1	7.4	1.1
**PP_RS13670**	PP_2622	*tssE*	4.7	1.5	4.8	1.5	1.0	1.8
**PP_RS13870**	PP_2663	*PP_2663*	4.7	1.0	5.2	1.0	9.0	1.0
**PP_RS13900**	PP_2669	*PP_2669*	4.4	1.1	4.7	1.1	8.1	1.0
**PP_RS13865**	PP_2662	*PP_2662*	4.4	1.5	4.9	1.5	7.3	1.5
**PP_RS13965**	PP_2681	*pqqD-II*	4.3	1.0	4.4	1.0	7.5	1.0
**PP_RS12795**	PP_2453	*PP_2453*	4.3	0.2	4.3	0.2	2.7	0.2
**PP_RS13905**	PP_5538	*PP_5538*	4.2	1.0	4.5	1.0	7.8	1.0
**PP_RS23955**	PP_4600	*moaB-II*	4.2	0.3	4.3	0.3	–0.2	0.4
**PP_RS12250**	PP_2349	*PP_2349*	4.2	0.4	4.1	0.4	0.6	0.4
**PP_RS00800**	PP_5747	*PP_5747*	3.8	0.3	3.8	0.3	0.5	0.3
**PP_RS01355**	PP_0257	*fdhD*	3.8	0.3	3.7	0.3	0.7	0.3
**PP_RS22955**	PP_4422	*PP_4422*	3.8	0.5	4.0	0.5	0.0	0.6
**PP_RS13675**	PP_2623	*tssC*	3.8	0.9	3.8	0.9	1.0	1.1
**PP_RS08990**	PP_1749	*ngg*	3.7	0.5	3.8	0.5	1.6	0.6
**PP_RS08995**	PP_1750	*PP_1750*	3.7	0.4	3.7	0.4	1.6	0.5
**PP_RS10880**	PP_5502	*PP_5502*	3.7	0.3	4.0	0.3	0.4	0.3
**PP_RS13895**	PP_2668	*PP_2668*	3.7	1.0	3.9	1.0	7.3	1.0
**PP_RS19820**	PP_3808	*PP_3808*	3.7	0.5	4.0	0.5	1.1	0.5
**PP_RS13945**	PP_2678	*PP_2678*	3.7	0.9	4.0	0.9	7.2	0.9
**PP_RS00805**	PP_0156	*PP_0156*	3.7	0.3	3.7	0.3	0.0	0.3
**PP_RS19815**	PP_3807	*PP_3807*	3.6	0.4	3.6	0.4	0.8	0.5
**PP_RS00795**	PP_0155	*PP_0155*	3.5	0.3	3.6	0.3	0.3	0.3
**PP_RS04590**	PP_0868	*PP_0868*	3.4	0.2	3.3	0.2	–0.9	0.2
**PP_RS21395**	PP_4116	*aceA*	3.2	0.9	3.5	0.9	4.7	0.9
**PP_RS19760**	PP_3796	*PP_3796*	3.2	0.5	3.3	0.5	0.7	0.5
**PP_RS21915**	PP_4221	*PP_4221*	3.2	0.6	3.3	0.6	0.2	0.7
**PP_RS19780**	PP_3800	*PP_3800*	3.2	0.4	3.1	0.4	1.5	0.4
**PP_RS19775**	PP_3799	*PP_3799*	3.2	0.4	3.3	0.4	1.3	0.4

**PP_RS19790**	PP_3802	*PP_3802*	3.2	0.4	3.1	0.4	1.7	0.5
**PP_RS12090**	PP_2315	*greB*	3.2	0.3	3.2	0.3	0.1	0.3
**PP_RS19770**	PP_3798	*PP_3798*	3.2	0.4	3.1	0.4	1.4	0.4
**PP_RS21910**	PP_4220	*PP_4220*	3.1	0.5	3.2	0.5	0.2	0.6
**PP_RS04595**	PP_0869	*PP_0869*	3.1	0.2	3.0	0.2	–1.3	0.3
**PP_RS01350**	PP_0256	*PP_0256*	3.1	0.2	3.2	0.2	0.5	0.2
**PP_RS13890**	PP_2667	*PP_2667*	3.1	1.0	3.4	1.0	6.5	1.0
**PP_RS19785**	PP_3801	*PP_3801*	3.1	0.4	3.1	0.4	1.6	0.4
**PP_RS19505**	PP_3747	*glcF*	3.0	0.8	3.0	0.8	–1.0	1.0
**PP_RS21895**	PP_4217	*PP_4217*	3.0	0.4	3.1	0.4	1.4	0.4
**PP_RS08985**	PP_1748	*PP_1748*	3.0	0.5	3.2	0.5	0.8	0.6
**PP_RS00815**	PP_0158	*PP_0158*	2.9	0.5	3.1	0.5	0.0	0.5
**PP_RS24710**	PP_4738	*PP_4738*	2.9	0.6	3.0	0.6	2.6	0.7
**PP_RS22040**	PP_4243	*PP_4243*	2.9	0.6	2.9	0.6	–0.1	0.8
**PP_RS13875**	PP_2664	*PP_2664*	2.8	0.9	3.2	0.9	6.7	0.9
**PP_RS19765**	PP_3797	*PP_3797*	2.8	0.4	2.8	0.4	1.8	0.4
**PP_RS13915**	PP_2672	*PP_2672*	2.8	1.0	3.1	1.0	5.9	1.0
**PP_RS06135**	PP_1185	*PP_1185*	2.8	0.1	2.8	0.1	–1.1	0.1
**PP_RS04600**	PP_0870	*PP_0870*	2.8	0.2	2.6	0.2	–1.5	0.2
**PP_RS02025**	PP_0383	*PP_0383*	2.8	0.2	2.9	0.2	0.1	0.2
**PP_RS13910**	PP_2671	*PP_2671*	2.7	0.9	2.9	0.9	6.1	0.9
**PP_RS11395**	PP_2197	*PP_2197*	2.7	0.3	2.5	0.3	1.3	0.3
**PP_RS06140**	PP_1186	*PP_1186*	2.7	0.1	2.5	0.1	–1.4	0.2
**PP_RS00705**	PP_0137	*gltP*	2.7	0.6	2.4	0.6	0.0	0.6
**PP_RS07665**	PP_1487	*PP_1487*	2.6	0.2	2.7	0.2	0.9	0.2
**PP_RS02875**	PP_0544	*eat*	2.6	0.4	2.7	0.4	–0.6	0.5
**PP_RS21905**	PP_4219	*PP_4219*	2.5	0.5	2.6	0.5	0.3	0.6
**PP_RS12085**	PP_2314	*PP_2314*	2.5	0.3	2.3	0.3	0.1	0.3
**PP_RS18870**	PP_3633	*argC*	2.5	0.3	2.3	0.3	–0.7	0.3
**PP_RS02880**	PP_0545	*PP_0545*	2.5	0.3	2.5	0.3	–0.8	0.3
**PP_RS19500**	PP_3746	*glcE*	2.4	0.7	2.5	0.7	–1.3	0.8
**PP_RS21870**	PP_4212	*PP_4212*	2.4	0.6	2.7	0.6	0.0	0.7
**PP_RS11200**	PP_2159	*PP_2159*	2.4	0.2	2.4	0.2	–0.2	0.3
**PP_RS25340**	PP_4855	*osmE*	2.4	0.6	2.2	0.6	2.8	0.6
**PP_RS06145**	PP_1187	*PP_1187*	2.4	0.2	2.4	0.2	–1.0	0.2
**PP_RS23940**	PP_4597	*moaA*	2.4	0.1	2.4	0.1	0.9	0.2
**PP_RS21920**	PP_4222	*PP_4222*	2.3	0.4	2.5	0.4	0.3	0.5
**PP_RS11400**	PP_2198	*PP_2198*	2.3	0.3	2.3	0.3	1.4	0.3
**PP_RS20580**	PP_3957	*betT-III*	2.3	0.1	2.6	0.1	–0.3	0.2
**PP_RS07745**	PP_1503	*PP_1503*	2.3	0.5	2.1	0.5	2.0	0.5
**PP_RS21885**	PP_4215	*PP_4215*	2.3	0.4	2.4	0.4	0.1	0.5
**PP_RS23930**	PP_4595	*PP_4595*	2.3	0.2	2.3	0.2	0.2	0.3
**PP_RS02865**	PP_0542	*eutC*	2.2	0.3	2.2	0.3	0.4	0.3

**PP_RS20600**	PP_3961	*PP_3961*	2.2	0.5	2.2	0.5	–0.4	0.6
**PP_RS13925**	PP_2674	*exaA*	2.2	1.1	2.7	1.1	6.3	1.1
**PP_RS04280**	PP_0806	*PP_0806*	2.2	0.4	2.2	0.4	0.1	0.4
**PP_RS02870**	PP_0543	*PP_0543*	2.2	0.4	2.2	0.4	0.0	0.4
**PP_RS00435**	PP_0085	*PP_0085*	2.2	0.4	2.3	0.4	2.1	0.4
**PP_RS02610**	PP_0493	*selA*	2.2	0.2	1.1	0.2	–0.2	0.2
**PP_RS19795**	PP_3803	*PP_3803*	2.1	0.3	2.3	0.3	1.0	0.4
**PP_RS22075**	PP_4250	*ccoN*	2.1	0.3	2.0	0.3	–1.3	0.3
**PP_RS04660**	PP_0883	*PP_0883*	2.1	0.3	2.1	0.3	–2.4	0.4
**PP_RS04605**	PP_0871	*PP_0871*	2.1	0.1	2.1	0.1	–1.4	0.2
**PP_RS20575**	PP_3956	*PP_3956*	2.1	0.2	2.2	0.2	–0.8	0.3
**PP_RS07025**	PP_1359	*fxsA*	2.1	0.2	2.1	0.2	1.0	0.2
**PP_RS12410**	PP_2380	*PP_2380*	2.1	0.1	1.8	0.1	0.1	0.2
**PP_RS26085**	PP_5000	*hslV*	2.0	0.2	2.0	0.2	1.0	0.2
**PP_RS02000**	PP_0378	*pqqC*	2.0	0.4	2.1	0.4	4.0	0.4
**PP_RS26445**	PP_5073	*PP_5073*	1.9	0.4	1.9	0.4	3.4	0.4
**PP_RS01435**	PP_0273	*PP_0273*	1.9	0.5	2.3	0.5	–1.0	0.6
**PP_RS13880**	PP_2665	*PP_2665*	1.9	1.0	1.7	1.0	4.2	1.0
**PP_RS25415**	PP_4870	*azu*	1.7	0.3	1.8	0.3	2.9	0.3
**PP_RS13885**	PP_2666	*PP_2666*	1.7	0.9	2.0	0.9	4.5	0.9
**PP_RS02005**	PP_0379	*pqqB*	1.6	0.3	1.7	0.3	3.3	0.3
**PP_RS27800**	PP_5338	*aspA*	1.6	0.1	1.5	0.1	3.6	0.1
**PP_RS13920**	PP_2673	*PP_2673*	1.5	1.1	1.9	1.1	5.7	1.1
**PP_RS01990**	PP_0376	*pqqE*	1.3	0.3	1.5	0.3	3.4	0.3
**PP_RS22095**	PP_4255	*ccoN.1*	1.3	0.3	1.5	0.3	2.9	0.3
**PP_RS12620**	PP_2422	*PP_2422*	1.3	0.7	1.5	0.7	3.4	0.7
**PP_RS22100**	PP_4256	*ccoO-II*	1.2	0.4	1.3	0.4	3.0	0.4
**PP_RS04220**	PP_0795	*PP_0795*	1.2	0.2	1.3	0.2	–2.1	0.3
**PP_RS22105**	PP_4257	*PP_4257*	1.1	0.4	1.2	0.4	3.1	0.4
**PP_RS11010**	PP_2121	*PP_2121*	1.1	0.1	1.6	0.1	2.2	0.1
**PP_RS01985**	PP_0375	*PP_0375*	1.0	0.3	1.3	0.3	3.6	0.3
**PP_RS04215**	PP_0794	*pfkB*	1.0	0.2	1.1	0.2	–2.3	0.3
**PP_RS26275**	PP_5038	*PP_5038*	1.0	0.5	0.9	0.5	2.8	0.5
**PP_RS24035**	PP_4614	*PP_4614*	1.0	0.3	1.0	0.3	2.7	0.3
**PP_RS13510**	PP_2589	*PP_2589*	1.0	0.8	0.8	0.8	2.9	0.7
**PP_RS04075**	PP_0763	*PP_0763*	1.0	0.2	1.0	0.2	2.3	0.2
**PP_RS04555**	PP_0861	*PP_0861*	0.9	1.1	0.7	1.1	6.6	1.1
**PP_RS01995**	PP_0377	*pqqD*	0.9	0.3	1.0	0.3	3.3	0.3
**PP_RS04210**	PP_0793	*ptsP*	0.8	0.2	0.9	0.2	–2.7	0.2
**PP_RS09400**	PP_1829	*PP_1829*	0.8	0.5	0.8	0.5	3.9	0.5
**PP_RS22110**	PP_4258	*ccoP-II*	0.8	0.3	1.0	0.3	2.6	0.3
**PP_RS00515**	PP_0101	*PP_0101*	0.8	0.2	0.9	0.2	2.4	0.2
**PP_RS08505**	PP_1651	*PP_1651*	0.6	0.6	0.8	0.6	2.4	0.6

**PP_RS24115**	PP_5702	*PP_5702*	0.4	0.2	0.4	0.2	2.1	0.2
**PP_RS01540**	PP_0288	*PP_0288*	0.4	0.3	0.6	0.2	2.4	0.2
**PP_RS11715**	PP_2256	*PP_2256*	0.3	0.4	0.4	0.4	2.3	0.4
**PP_RS18625**	PP_3585	*PP_3585*	0.3	0.2	0.3	0.2	3.0	0.2
**PP_RS01150**	PP_0216	*PP_0216*	0.3	0.4	0.4	0.4	2.5	0.4
**PP_RS14245**	PP_2737	*PP_2737*	0.2	0.5	0.0	0.5	2.6	0.5
**PP_RS18620**	PP_3584	*PP_3584*	0.2	0.2	0.2	0.2	2.5	0.2
**PP_RS14240**	PP_2736	*PP_2736*	0.2	0.5	0.1	0.5	2.3	0.5
**PP_RS13505**	PP_2588	*PP_2588*	0.2	0.5	0.3	0.5	2.2	0.4
**PP_RS20450**	PP_3929	*PP_3929*	0.1	0.4	–0.4	0.4	2.8	0.3
**PP_RS14250**	PP_2738	*PP_2738*	0.1	0.5	0.1	0.5	2.3	0.4
**PP_RS04560**	PP_0862	*PP_0862*	0.1	0.7	0.0	0.7	2.6	0.6
**PP_RS04550**	PP_0860	*PP_0860*	0.0	0.5	–0.1	0.5	2.5	0.5
**PP_RS21505**	PP_4139	*PP_4139*	–0.1	0.2	–0.3	0.2	2.1	0.2
**PP_RS07315**	PP_1416	*PP_1416*	–0.2	0.4	–0.7	0.4	2.2	0.3
**PP_RS27045**	PP_5191	*PP_5191*	–0.3	0.4	–0.2	0.3	2.2	0.3
**PP_RS18610**	PP_3582	*PP_3582*	–0.3	0.2	–0.3	0.2	2.2	0.2
**PP_RS18615**	PP_3583	*PP_3583*	–0.3	0.2	–0.1	0.2	2.1	0.2
**PP_RS11045**	PP_5503	*PP_5503*	–0.3	0.2	–0.4	0.2	–2.6	0.2
**PP_RS17565**	PP_3372	*PP_3372*	–0.4	0.4	–0.6	0.4	2.6	0.3
**PP_RS05610**	PP_1076	*PP_1076*	–0.4	0.2	–0.4	0.2	–2.1	0.2
**PP_RS27320**	PP_5241	*PP_5241*	–0.5	0.4	–0.3	0.4	2.2	0.3
**PP_RS25920**	PP_4967	*metK*	–0.5	0.1	–0.6	0.1	–2.4	0.1
**PP_RS23455**	PP_4505	*PP_4505*	–0.6	0.2	–0.8	0.2	3.1	0.2
**PP_RS05605**	PP_1075	*glpK*	–0.6	0.1	–0.6	0.1	–2.0	0.1
**PP_RS00260**	PP_0050	*PP_0050*	–0.6	0.3	–0.6	0.3	4.5	0.2
**PP_RS01980**	PP_5444	*PP_5444*	–0.7	0.3	–0.2	0.3	2.6	0.3
**PP_RS19615**	PP_3769	*PP_3769*	–0.7	0.3	–0.7	0.3	3.5	0.2
**PP_RS23450**	PP_4504	*PP_4504*	–0.8	0.2	–0.8	0.2	6.8	0.2
**PP_RS19670**	PP_3779	*PP_3779*	–0.9	0.1	–0.5	0.1	–2.5	0.2
**PP_RS25915**	PP_4966	*PP_4966*	–0.9	0.1	–1.1	0.1	–2.9	0.1
**PP_RS19620**	PP_3770	*PP_3770*	–1.0	0.9	–0.1	0.8	3.8	0.7
**PP_RS19660**	PP_3777	*PP_3777*	–1.0	0.2	–0.6	0.1	–4.4	0.2
**PP_RS19665**	PP_3778	*PP_3778*	–1.1	0.2	–0.6	0.2	–4.6	0.2
**PP_RS19655**	PP_3776	*PP_3776*	–1.2	0.1	–0.9	0.1	–3.4	0.2
**PP_RS19650**	PP_3775	*solA*	–1.4	0.1	–1.1	0.1	–3.1	0.1
**PP_RS07325**	PP_1418	*PP_1418*	–1.4	0.5	–1.5	0.5	2.5	0.4
**PP_RS23685**	PP_4547	*PP_4547*	–1.5	0.3	–1.7	0.3	–3.6	0.3
**PP_RS23690**	PP_4548	*PP_4548*	–1.6	0.2	–1.8	0.2	–4.1	0.3
**PP_RS21410**	PP_4120	*PP_4120*	–1.8	0.1	–2.1	0.1	0.0	0.1
**PP_RS19345**	PP_3715	*PP_3715*	–1.9	0.6	–1.8	0.6	–2.4	0.6
**PP_RS08700**	PP_1692	*PP_1692*	–1.9	0.3	–2.3	0.4	–1.9	0.3
**PP_RS21420**	PP_4122	*nuoE*	–2.0	0.1	–2.2	0.1	0.2	0.1

**PP_RS21425**	PP_4123	*nuoF*	–2.0	0.1	–2.2	0.1	0.1	0.1
**PP_RS09570**	PP_1864	*PP_1864*	–2.0	0.4	–1.7	0.4	–4.6	0.7
**PP_RS25970**	PP_4977	*metF*	–2.1	0.1	–2.1	0.1	–2.2	0.1
**PP_RS25965**	PP_4976	*ahcY*	–2.1	0.1	–2.2	0.1	–2.2	0.1
**PP_RS13175**		*glsB**	–2.1	0.2	–2.1	0.2	0.2	0.2
**PP_RS11650**	PP_2243	*PP_2243*	–2.2	0.2	–2.3	0.2	–1.5	0.2
**PP_RS21430**	PP_4124	*nuoG*	–2.2	0.1	–2.4	0.1	0.0	0.1
**PP_RS21435**	PP_4125	*nuoH*	–2.2	0.1	–2.3	0.1	0.0	0.1
**PP_RS07330**	PP_1419	*PP_1419*	–2.2	0.5	–3.0	0.6	2.9	0.5
**PP_RS12760**	PP_2448	*PP_2448*	–2.2	0.2	–2.2	0.2	–2.8	0.2
**PP_RS19680**	PP_3782	*PP_3782*	–2.2	0.3	–1.8	0.3	–9.2	0.6
**PP_RS24860**	PP_5712	*PP_5712*	–2.3	0.4	–2.5	0.4	1.8	0.3
**PP_RS19685**	PP_3783	*PP_3783*	–2.3	0.1	–2.1	0.1	–8.7	0.2
**PP_RS07145**	PP_1383	*PP_1383*	–2.3	0.4	–2.4	0.4	–1.4	0.3
**PP_RS07800**	PP_1514	*PP_1514*	–2.3	0.3	–1.8	0.3	–0.5	0.3
**PP_RS21440**	PP_4126	*nuoI*	–2.3	0.1	–2.7	0.1	0.0	0.1
**PP_RS13460**	PP_2579	*PP_2579*	–2.3	0.4	–2.3	0.4	0.4	0.3
**PP_RS03135**	PP_0596	*PP_0596*	–2.3	0.3	–2.3	0.3	–2.8	0.3
**PP_RS18405**	PP_3541	*PP_3541*	–2.4	0.3	–2.1	0.3	–0.5	0.2
**PP_RS21450**	PP_4128	*nuoK*	–2.4	0.1	–2.5	0.1	–0.1	0.1
**PP_RS19690**	PP_3784	*PP_3784*	–2.4	0.1	–2.1	0.1	–8.2	0.3
**PP_RS00290**	PP_0056	*PP_0056*	–2.4	0.3	–2.6	0.3	–2.0	0.3
**PP_RS21735**	PP_4186	*sucC*	–2.4	0.1	–2.4	0.1	–0.1	0.1
**PP_RS21730**	PP_4185	*sucD*	–2.4	0.1	–2.3	0.1	–0.2	0.1
**PP_RS19675**	PP_3781	*PP_3781*	–2.4	0.1	–2.0	0.1	–9.4	0.3
**PP_RS23915**	PP_4592	*PP_4592*	–2.4	0.2	–2.5	0.2	–0.4	0.2
**PP_RS21455**	PP_4129	*nuoL*	–2.5	0.1	–2.5	0.1	0.0	0.1
**PP_RS04760**	PP_0904	*PP_0904*	–2.5	0.3	–2.5	0.3	1.5	0.2
**PP_RS21465**	PP_4131	*nuoN*	–2.5	0.1	–2.5	0.1	–0.2	0.1
**PP_RS21460**	PP_4130	*nuoM*	–2.5	0.1	–2.5	0.1	–0.1	0.1
**PP_RS21445**	PP_4127	*nuoJ*	–2.6	0.1	–2.7	0.1	–0.1	0.1
**PP_RS19695**	PP_3785	*PP_3785*	–2.6	0.1	–2.2	0.1	–7.8	0.2
**PP_RS16510**	PP_3166	*catA*	–2.7	0.6	–3.1	0.6	–2.7	0.6
**PP_RS19700**	PP_3786	*PP_3786*	–2.8	0.1	–2.6	0.1	–6.8	0.2
**PP_RS19705**	PP_3787	*PP_3787*	–2.8	0.1	–2.6	0.1	–6.2	0.2
**PP_RS19725**	PP_3790	*dapF*	–2.9	0.2	–2.7	0.1	–4.7	0.2
**PP_RS19710**	PP_3788	*PP_3788*	–2.9	0.2	–2.6	0.2	–6.4	0.2
**PP_RS19720**	PP_3789	*PP_3789*	–2.9	0.1	–2.6	0.1	–5.3	0.2
**PP_RS19715**	PP_5628	*PP_5628*	–3.0	0.2	–2.6	0.2	–6.5	0.3
**PP_RS19730**	PP_3791	*PP_3791*	–3.0	0.3	–2.5	0.3	–3.0	0.3
**PP_RS16505**	PP_3165	*PP_3165*	–3.1	0.7	–3.4	0.7	–2.7	0.6
**PP_RS04755**	PP_0903	*PP_0903*	–3.5	0.3	–3.4	0.3	1.5	0.2
**PP_RS17600**	PP_3379	*PP_3379*	–3.5	0.2	–2.8	0.1	0.6	0.1

**PP_RS17570**	PP_3373	*bamA-II*	–3.7	0.4	–3.2	0.4	0.0	0.2
**PP_RS16500**	PP_3164	*PP_3164*	–3.8	0.6	–4.0	0.6	–4.2	0.6
**PP_RS25035**	PP_4797	*arfA*	–4.1	1.2	–4.2	1.2	–1.2	0.7
**PP_RS17595**	PP_3378	*PP_3378*	–4.3	0.2	–3.3	0.2	0.4	0.2
**PP_RS17585**	PP_3376	*PP_3376*	–4.6	0.2	–3.8	0.1	0.3	0.1
**PP_RS17590**	PP_3377	*PP_3377*	–4.7	0.2	–3.6	0.2	0.5	0.2
**PP_RS17575**	PP_3374	*PP_3374*	–4.7	0.2	–3.8	0.1	0.0	0.1
**PP_RS16490**	PP_3162	*benB*	–4.8	0.4	–5.2	0.5	–4.0	0.4
**PP_RS07225**	PP_1400	*PP_1400*	–5.0	0.3	–4.8	0.3	0.3	0.1
**PP_RS16485**	PP_3161	*benA*	–5.4	0.4	–5.3	0.4	–4.2	0.3
**PP_RS16495**	PP_3163	*PP_3163*	–5.4	0.6	–5.8	0.7	–5.0	0.6

The table represents significant changes in gene expression with a *q*-value threshold of 0.01. Log_2(FC) and standard-error (SE) are indicated for each comparison to the control group (*P. putida* EM42 cultivated in DBM medium supplemented with 20 mM glucose). The asterisk symbol (*) indicates a pseudogene.
