# Supplementary material for: Core and auxiliary functions of one-carbon metabolism in Pseudomonas putida exposed by a systems-level analysis of transcriptional and physiological responses
Source: mSystems. 2023 Jun 5;8(3):e00004-23. doi: 10.1128/msystems.00004-23 (PMC10308882; doi:10.1128/msystems.00004-23)
Supplement: TABLE S2 — Gene ontology analysis for P. putida EM42 genes differentially expressed in the methanol and formate conditions compared to the glucose-only control. [file msystems.00004-23-s0007.pdf]

Table S2. Gene ontology analysis for *P. putida* EM42 genes differentially expressed in the methanol and formate conditions compared to the glucose-only control.

Gene ontology · Terms	*P*-values	Matches
EM42 formate *versus* EM42 glucose		
NADH dehydrogenase (quinone) activity	2.78×10^{-12}	*nuoN · nuoM · nuoL · nuoJ · nuoG · nuoF · nuoB · nuoK · nuoI*
NAD(P)H dehydrogenase (quinone) activity	1.86×10^{-11}	*nuoN · nuoM · nuoL · nuoJ · nuoG · nuoF · nuoB · nuoK · nuoI*
NADH dehydrogenase activity	1.86×10^{-12}	*nuoN · nuoM · nuoL · nuoJ · nuoG · nuoF · nuoB · nuoK · nuoI*
Oxidoreductase activity	4.76×10^{-10}	*nuoM · pedH · nuoJ · kguD · nuoG · glcF · cioA · PP_4596 · benD · nuoE · PP_0256 · benB · benA · pqqC · nuoB · yliI · pvdA · nuoN · argC · PP_2052 · metF · pedI · kguE · pntB · nuoL · nuoK · davB · aldB-I · nuoI · gabD-II · nuoH · pedF · gcdH · pntA · cinA · puuB · nuoF · benC · betA-I · glcE · PP_4222*
NADH dehydrogenase (ubiquinone) activity	8.06×10^{-10}	*nuoN · nuoM · nuoL · nuoJ · nuoG · nuoF · nuoB*
Oxidoreductase activity, acting on NAD(P)H, quinone or similar compound as acceptor	8.99×10^{-10}	*nuoN · nuoM · nuoL · nuoJ · nuoG · nuoF · nuoB · nuoK · nuoI · nuoH*
Quinone binding	9.69×10^{-10}	*nuoN · nuoK · nuoI · nuoH · nuoG · nuoF · nuoB · nuoJ*
Oxidoreductase activity, acting on NAD(P)H	1.61×10^{-9}	*pntB · nuoN · nuoM · nuoL · nuoJ · nuoG · nuoF · nuoB · nuoK · nuoI · nuoH*
Oxidoreduction-driven active transmembrane transporter activity	6.16×10^{-7}	*nuoN · nuoM · nuoL · nuoJ · nuoG · nuoF · nuoB*
Phosphopantetheine binding	2.24×10^{-6}	*pvdL · pvdI · pvdJ · pvdD*
Modified amino acid binding	2.24×10^{-6}	*pvdL · pvdI · pvdJ · pvdD*
Iron-sulfur cluster binding	1.61×10^{-5}	*nuoG · glcF · PP_4596 · nuoE · PP_0256 · benA · nuoB · PP_3781 · nuoI · moaA · nuoF · benC*
Metal cluster binding	1.61×10^{-5}	*nuoG · glcF · PP_4596 · nuoE · PP_0256 · benA · nuoB · PP_3781 · nuoI · moaA · nuoF · benC*
Benzoate 1,2-dioxygenase activity	1.79×10^{-5}	*benC · benB · benA*
Electron transfer activity	3.68×10^{-5}	*nuoN · nuoM · nuoL · nuoJ · nuoG · nuoF · nuoB · cioA · benC · pedF · cinA*
4Fe, 4S cluster binding	6.72×10^{-5}	*moaA · PP_4596 · nuoI · nuoG · nuoF · nuoB · PP_0256 · glcF*
Oxidoreductase activity, acting on paired donors, with incorporation or reduction of molecular oxygen, NAD(P)H as one donor, and incorporation of two atoms of oxygen into one donor	7.02×10^{-5}	*benC · benB · benA*
Amide binding	1.31×10^{-4}	*pvdL · pvdI · pvdJ · pvdD*
Succinate-CoA ligase (ADP-forming) activity	6.91×10^{-4}	*sucC · sucD*
Ethanolamine ammonia-lyase activity	6.91×10^{-4}	*eutB · eutC*
Succinate-CoA ligase activity	6.91×10^{-4}	*sucC · sucD*
Primary active transmembrane transporter activity	0.0011974	*nuoN · nuoM · nuoL · nuoJ · nuoG · nuoF · nuoB · pedC · fpvE*
Oxidoreductase activity, acting on the aldehyde or oxo group of donors, NAD or NADP as acceptor	0.0016201	*pedI · aldB-I · gabD-II · argC · PP_4596 · PP_0256*
Glycolate dehydrogenase activity	0.0020387	*glcF · glcE*
Oxidoreductase activity, acting on the aldehyde or oxo group of donors	0.0029578	*pedI · aldB-I · gabD-II · argC · PP_4596 · PP_0256*
Glyceraldehyde-3-phosphate dehydrogenase (NAD^+) (non-phosphorylating) activity	0.0040072	*pedI · aldB-I*

Active transmembrane transporter activity	0.0042087	*gltP · nuoN · nuoM · nuoL · nuoJ · nuoG · nuoF · nuoB · pedC · fpvE*
Catalytic activity	0.0045766	*pvdJ · nuoM · pedH · yehX · PP_3807 · nuoJ · kguD · sucD · eutB · nuoG · glcF · cioA · PP_4596 · benD · fpvD · nuoE · pedS2 · PP_0256 · benB · phoQ · benA · pqqC · aspC · nuoB · pvdI · yliI · aceA · pvdA · nuoN · asnB · argC · PP_2052 · metF · pedI · kguE · fdhD · ahcY · pvdL · pvdD · pntB · ansB · nuoL · PP_3781 · nuoK · PP_1748 · kguK · pedS1 · PP_2678 · davB · aldB-I · sucC · nuoI · gabD-II · nuoH · pedF · gcdH · moaA · pntA · cinA · eutC · puuB · nuoF · opdH · benC · betA-I · PP_3787 · glcE · PP_4592 · PP_4222 · PP_0596 · yijP*
Formate dehydrogenase (NAD^+) activity	0.006564	*PP_4596 · PP_0256*
Molybdenum ion binding	0.006564	*PP_4596 · PP_0256*
Vitamin binding	0.0070418	*aspC · PP_0596 · eutC · pvdL · pvdI · pvdJ · pvdD*
Oxidoreductase activity, acting on CH-OH group of donors	0.015567	*glcF · glcE · kguE · PP_2052 · kguD · betA-I · pedH*
2Fe, 2S cluster binding	0.0202128	*nuoG · nuoE · benC · benA*
Succinate-semialdehyde dehydrogenase ($NADP^+$) activity	0.0264381	*gabD-II*
Oxidoreductase activity, acting on phosphorus or arsenic in donors, with $NAD(P)^+$ as acceptor	0.0264381	*kguD*
Ferredoxin-NAD^+ reductase activity	0.0264381	*benC*
Phosphonate dehydrogenase activity	0.0264381	*kguD*
GTP 3',8'-cyclase activity	0.0264381	*moaA*
Adenosylhomocysteinase activity	0.0264381	*ahcY*
Lysine 2-monooxygenase activity	0.0264381	*davB*
Asparagine synthase (glutamine-hydrolyzing) activity	0.0264381	*asnB*
Pyrroloquinoline-quinone synthase activity	0.0264381	*pqqC*
β-Alanine-pyruvate transaminase activity	0.0264381	*PP_0596*
L-Aspartate:2-oxoglutarate aminotransferase activity	0.0264381	*aspC*
Hydrolase activity, acting on ether bonds	0.0264381	*ahcY*
$NAD(P)^+$ transhydrogenase (AB-specific) activity	0.0264381	*pntB*
Phosphogluconate 2-dehydrogenase activity	0.0264381	*kguE*
Isocitrate lyase activity	0.0264381	*aceA*
Amino acid:proton symporter activity	0.0264381	*gltP*
Trialkylsulfonium hydrolase activity	0.0264381	*ahcY*
Dehydrogluconokinase activity	0.0264381	*kguK*
Glutamin-(asparagin-)ase activity	0.0264381	*ansB*
1,6-dihydroxycyclohexa-2,4-diene-1-carboxylate dehydrogenase activity	0.0264381	*benD*
D-2-hydroxy-acid dehydrogenase activity	0.0264381	*glcF*
Glutaryl-CoA dehydrogenase activity	0.0264381	*gcdH*
Mannitol-1-phosphate 5-dehydrogenase activity	0.0264381	*PP_2052*
Sulfuric ester hydrolase activity	0.0271006	*PP_4592 · yijP*

Acid-thiol ligase activity	0.0271006	*sucC · sucD*
Protein dimerization activity	0.0384113	*pedS2 · argC*
Aldehyde dehydrogenase (NAD+) activity	0.0384113	*pedI · aldB-I*
Ammonia-lyase activity	0.044628	*eutB · eutC*
Ligase activity, forming carbon-sulfur bonds	0.044628	*sucC · sucD*
EM42 ΔΔFDH formate *versus* EM42 glucose		
ATP synthesis coupled electron transport	4.81×10^{-9}	*nuoN · nuoM · nuoL · nuoK · nuoG*
Respiratory electron transport chain	1.13×10^{-8}	*cioA · nuoN · nuoM · nuoL · nuoK · nuoG · fdoI*
Electron transport chain	1.25×10^{-6}	*cioA · nuoN · nuoM · nuoL · nuoK · nuoG · fdoI · benC · pedF · cinA · fmdF · nuoJ*
Generation of precursor metabolites and energy	2.03×10^{-5}	*cioA · nuoN · nuoM · nuoL · nuoK · nuoG · fdoI · benC · pedF · cinA · fmdF · nuoJ*
Asparagine metabolic process	0.001461	*asnB · ansB*
Secondary metabolite biosynthetic process	0.0047303	*pvdD · pvdL*
Secondary metabolic process	0.0069933	*pvdD · pvdL*
Ethanolamine-containing compound metabolic process	0.022404	*eutC*
Primary amino compound catabolic process	0.022404	*eutC*
Glycine betaine transport	0.022404	*yehX*
Ethanolamine metabolic process	0.022404	*eutC*
Amino-acid betaine transport	0.022404	*yehX*
Asparagine biosynthetic process	0.022404	*asnB*
Primary alcohol catabolic process	0.022404	*eutC*
Modified amino acid transport	0.022404	*yehX*
Quaternary ammonium group transport	0.022404	*yehX*
Ethanolamine catabolic process	0.022404	*eutC*
Primary amino compound metabolic process	0.022404	*eutC*
Molybdopterin cofactor metabolic process	0.0238563	*moaA · fdhD*
prosthetic group metabolic process	0.0238563	*moaA · fdhD*
Mo-molybdopterin cofactor biosynthetic process	0.0238563	*moaA · fdhD*
Mo-molybdopterin cofactor metabolic process	0.0238563	*moaA · fdhD*
One-carbon metabolic process	0.0378091	*metF · ahcY*
Cellular respiration	0.0378091	*cioA · fdoI*
Metabolic process	0.0381723	*pvdJ · nuoM · PP_3807 · nuoJ · sucD · eutB · nuoG · cioA · pedS2 · benB · phoQ · benA · aspC · pvdI · aceA · selA · pvdA · nuoN · asnB · argC · metF · pedI · fdhD · ahcY · pvdL · pvdD · ansB · nuoL · nuoK · PP_1748 · kguK · aldB-I · sucC · gabD-II · PP_3791 · fdoI · pedF · hslV · moaA · cinA · eutC · benC · PP_3787 · PP_0596 · fmdF*
Regulation of cellular macromolecule biosynthetic process	0.0421649	*selA · rmf · ybaO*
Cellular biogenic amine catabolic process	0.0443138	*eutC*
Translational readthrough	0.0443138	*selA*

Selenocysteinyl-tRNA(Sec) biosynthetic process	0.0443138	*selA*
Amine catabolic process	0.0443138	*eutC*
Selenocysteine incorporation	0.0443138	*selA*
ATP synthesis coupled electron transport	4.81×10^{-9}	*nuoN · nuoM · nuoL · nuoK · nuoG*
Respiratory electron transport chain	1.13×10^{-8}	*cioA · nuoN · nuoM · nuoL · nuoK · nuoG · fdoI*
Electron transport chain	1.25×10^{-6}	*cioA · nuoN · nuoM · nuoL · nuoK · nuoG · fdoI · benC · pedF · cinA · fmdF · nuoJ*
Generation of precursor metabolites and energy	2.03×10^{-5}	*cioA · nuoN · nuoM · nuoL · nuoK · nuoG · fdoI · benC · pedF · cinA · fmdF · nuoJ*
Asparagine metabolic process	0.001461	*asnB · ansB*
Secondary metabolite biosynthetic process	0.0047303	*pvdD · pvdL*
Secondary metabolic process	0.0069933	*pvdD · pvdL*
Ethanolamine-containing compound metabolic process	0.022404	*eutC*
Primary amino compound catabolic process	0.022404	*eutC*
Glycine betaine transport	0.022404	*yehX*
Ethanolamine metabolic process	0.022404	*eutC*
Amino-acid betaine transport	0.022404	*yehX*
Asparagine biosynthetic process	0.022404	*asnB*
Primary alcohol catabolic process	0.022404	*eutC*
Modified amino acid transport	0.022404	*yehX*
Quaternary ammonium group transport	0.022404	*yehX*
Ethanolamine catabolic process	0.022404	*eutC*
Primary amino compound metabolic process	0.022404	*eutC*
Molybdopterin cofactor metabolic process	0.0238563	*moaA · fdhD*
Prosthetic group metabolic process	0.0238563	*moaA · fdhD*
Mo-molybdopterin cofactor biosynthetic process	0.0238563	*moaA · fdhD*
Mo-molybdopterin cofactor metabolic process	0.0238563	*moaA · fdhD*
One-carbon metabolic process	0.0378091	*metF · ahcY*
Cellular respiration	0.0378091	*cioA · fdoI*
Metabolic process	0.0381723	*pvdJ · nuoM · PP_3807 · nuoJ · sucD · eutB · nuoG · cioA · pedS2 · benB · phoQ · benA · aspC · pvdI · aceA · selA · pvdA · nuoN · asnB · argC · metF · pedI · fdhD · ahcY · pvdL · pvdD · ansB · nuoL · nuoK · PP_1748 · kguK · aldB-I · sucC · gabD-II · PP_3791 · fdoI · pedF · hslV · moaA · cinA · eutC · benC · PP_3787 · PP_0596 · fmdF*
Regulation of cellular macromolecule biosynthetic process	0.0421649	*selA · rmf · ybaO*
Cellular biogenic amine catabolic process	0.0443138	*eutC*
Translational read-through	0.0443138	*selA*
Selenocysteinyl-tRNA(Sec) biosynthetic process	0.0443138	*selA*
Amine catabolic process	0.0443138	*eutC*
Selenocysteine incorporation	0.0443138	*selA*

EM42 methanol *versus* EM42 glucose		
Benzoate 1,2-dioxygenase activity	8.42×10^{-6}	*benC* · *benB* · *benA*
Oxidoreductase activity, acting on paired donors, with incorporation or reduction of molecular oxygen, NAD(P)H as one donor, and incorporation of two atoms of oxygen into one donor	3.32×10^{-5}	*benC* · *benB* · *benA*
Dioxygenase activity	0.0043302	*benC* · *benB* · *benA* · *PP_0862*
Oxidoreductase activity, acting on paired donors, with incorporation or reduction of molecular oxygen	0.0079318	*benC* · *benB* · *benA* · *PP_0862*
D-Glucosamine PTS permease activity	0.0206275	*fruA*
Carbohydrate:cation symporter activity	0.0206275	*fruA*
Ferredoxin-NAD^+ reductase activity	0.0206275	*benC*
Hexose transmembrane transporter activity	0.0206275	*fruA*
Adenosylhomocysteinase activity	0.0206275	*ahcY*
Aspartate ammonia-lyase activity	0.0206275	*aspA*
Methionine adenosyltransferase activity	0.0206275	*metK*
Pyrroloquinoline-quinone synthase activity	0.0206275	*pqqC*
β-Alanine-pyruvate transaminase activity	0.0206275	*PP_0596*
L-Aspartate:2-oxoglutarate aminotransferase activity	0.0206275	*aspC*
Chloromuconate cycloisomerase activity	0.0206275	*catB*
Fructose transmembrane transporter activity	0.0206275	*fruA*
Hydrolase activity, acting on ether bonds	0.0206275	*ahcY*
Isocitrate lyase activity	0.0206275	*aceA*
Trialkylsulfonium hydrolase activity	0.0206275	*ahcY*
Glutamin-(asparagin-)ase activity	0.0206275	*ansB*
1,6-Dihydroxycyclohexa-2,4-diene-1-carboxylate dehydrogenase activity	0.0206275	*benD*
Muconate cycloisomerase activity	0.0206275	*catB*
Carbohydrate:proton symporter activity	0.0206275	*fruA*
Protein-N(PI)-phosphohistidine-fructose phosphotransferase system transporter activity	0.0206275	*fruA*
Glycerol kinase activity	0.0206275	*glpK*
Oxidoreductase activity	0.0306656	*PP_2737* · *pedH* · *benD* · *PP_2422* · *benB* · *PP_0860* · *benA* · *pqqC* · *PP_4548* · *metF* · *pedI* · *pqqE* · *PP_2736* · *PP_2589* · *proC* · *pedF* · *puuB* · *PP_0862* · *benC*
Vitamin binding	0.0310217	*PP_0862* · *aspC* · *PP_2588* · *PP_0596* · *PP_3782*
Oxidoreductase activity, acting on the CH-OH group of donors, cytochrome as acceptor	0.0408355	*pedH*
Methylenetetrahydrofolate reductase (NAD(P)H) activity	0.0408355	*metF*
Phosphoenolpyruvate-protein phosphotransferase activity	0.0408355	*ptsP*

Alcohol dehydrogenase (cytochrome *c*) activity	0.0408355	*pedH*
Solute:proton symporter activity	0.0408355	*fruA*
Protein-N(PI)-phosphohistidine-sugar phosphotransferase activity	0.0408355	*fruA*
Methylenetetrahydrofolate reductase NADPH activity	0.0408355	*metF*
Pyrroline-5-carboxylate reductase activity	0.0408355	*proC*
Cyclase activity	0.0408355	*pqqE*
Methylenetetrahydrofolate reductase NADH activity	0.0408355	*metF*
L-Ascorbic acid binding	0.0408355	*PP_0862*
Transaminase activity	0.0424008	*PP_0596 · aspC · PP_2588*
Transferase activity, transferring nitrogenous groups	0.0452732	*PP_0596 · aspC · PP_2588*
Oxidoreductase activity, acting on the CH-NH group of donors, NAD or NADP as acceptor	0.0467337	*proC · metF*

The table represents significant gene ontology (GO) terms detected from the differentially expressed genes [DEGs, i.e. gene expression with $|Log_2(FC)| \geq 2$ and a *P*-value ≤ 0.01]. *P*-values for the GO analysis are also given and threshold is set to 0.05. Genes represented in the different GO-terms are also given for each condition.
