# Supplementary material for: Core and auxiliary functions of one-carbon metabolism in Pseudomonas putida exposed by a systems-level analysis of transcriptional and physiological responses
Source: mSystems. 2023 Jun 5;8(3):e00004-23. doi: 10.1128/msystems.00004-23 (PMC10308882; doi:10.1128/msystems.00004-23)
Supplement: TABLE S3 — Oligonucleotides used in this study. [file msystems.00004-23-s0008.pdf]

Table S3. Oligonucleotides used in this study.

Name	DNA sequence (5'→3')	Use
PP0256_US_U_F	AGATCCUCAATGGCTGAAAGTGTGTCG	Construction of pGNW2·Δ*PP_0256-57*
PP0256_US_U_R	ATCGGGGCGCUGAATACCGGCGGCTTTCT	
PP0256_DS_U_F	AGCGCCCCGAUGTAAAAACGAAAAAAGCCCTG	
PP0256_DS_U_R	AGGTCGACUCAATGTAGCTACCCGTGG	
PP4596_US_U_F	AGATCCUCCGGGCAGGGCGGCGATG	Construction of pGNW2·Δ*PP_4596*
PP4596_US_U_R	ATCACAUGCGATGAATCCTCACGCTTCGATG	
PP4596_DS_U_F	ATGTGAUGGGGTGCCTGTGCTGGCC	
PP4596_DS_U_R	AGGTCGACUCGAGGCCGTCAACCTGTCC	
fdhA_US_U_F	AGATCCUCACTACATCAACAACGACCTGGAT	Construction of pGNW2·Δ*fdhA*
fdhA_US_U_R	ATTACAUGCTCTTACCTCGCTTGATTTTT	
fdhA_DS_U_F	ATGTAAUCAGCGTCGAGTAAAAAGAG	
fdhA_DS_U_R	AGGTCGACUCAAGAATTGACGCCGCTA	
fdhB_US_U_F	AGATCCUAGGTGTTGGTAGTAGAGG	Construction of pGNW2·Δ*fdhB*
fdhB_US_U_R	ACATAGUCTTGCTCCTGCAAAAAAA	
fdhB_DS_U_F	ACTATGUAGAGGAGGAATGATCATG	
fdhB_DS_U_R	AGGTCGACUTACCTGCCTCTTTGTTTG	
frmAC_US_U_F	AGATCCUGGTCGAGGGTGTGGTTGCT	Construction of pGNW2·Δ*frmAC*
frmAC_US_U_R	ACAAAGGUCTCCAGACAGAGGATCGATT	
frmAC_DS_U_F	ACCTTTGUAACGCCTCCTGTGGGAGC	
frmAC_DS_U_R	AGGTCGACUTGGTGGCCTTGACATTGACCT	
aldB-II_US_U_F	AGATCCUAACGAGTGGGGCATGGACA	Construction of pGNW2·Δ*aldB-II*
aldB-II_US_U_R	ACATGCUGAGCCTCTGCGGGTCGGT	
aldB-II_DS_U_F	AGCATGUAAACCGCGTCGCGGCCTT	
aldB-II_DS_U_R	AGGTCGACUCACCACGAAAGCAACCAGACCA	
adhP_US_U_F	AGATCCUCGTCTTCCTCCACTTCAT	Construction of pGNW2·Δ*adhP*
adhP_US_U_R	ATGAGAGCCUCCGTGTCTATCCGATGTG	
adhP_DS_U_F	AGGCTCTCAUGTGACAGGCCGAGCCAGGT	
adhP_DS_U_R	AGGTCGACUAGGGTCTGGTGGCAGGTTCT	
yiaY_US_U_F	AGATCCUGACTTGCCCTATGTGGACT	Construction of pGNW2·Δ*yiaY*
yiaY_US_U_R	ACATCGGUAAGCCTGTTCTTATTGTTCTG	
yiaY_DS_U_F	ACCGATGUGACGAGCAGCAGCGGGCG	
yiaY_DS_U_R	AGGTCGACUCAATTCATCGGTACGCGCAGCA	
pedE_US_U_F	AGATCCUAGCGCAGTTCTGGTTGTAG	Construction of pGNW2·Δ*pedE*
pedE_US_U_R	AGGTCACAUGGTTGCAGTTCCCAGTGG	
pedE_DS_U_F	ATGTGACCUGCAGCGGGGAGCGGCCTG	
pedE_DS_U_R	AGGTCGACUTGCAGCTTTGCCCCGGAC	
pedH_US_U_F	AGATCCUGATGAAAAGACCGGGGTGC	Construction of pGNW2·Δ*pedH*
pedH_US_U_R	AGGCGTTACAUGACGGCTACCTTTGGTTT	
pedH_DS_U_F	ATGTAACGCCUGCCTACTGCCGCTTGCTG	
pedH_DS_U_R	AGGTCGACUTTCAAGGTTTCGCGTATGG	
pS621_PP0256-57_F	ATCTAGAGUCGACCTGCAGGCATGCAA	Construction of pS621·*PP_0256-57*
pS621_PP0256-57_R	ATATGTUTTTCCTCCTGGGAATTCGC	

PP0256-57_ pS621_F	AACATAUGAACAGCAAGCCTCCGGT	
PP0256-57_ pS621_R	ACTCTAGAUTAAAGGATACGCCCGTCCT	
pS621_eV_F	ATATGTAAUCTAGAGTCGACCTGCAGG	Construction of pS621c
pS621_eV_R	ATTACATAUGTTTTTCCTCCTGGGAAT	
